# The role of network connectivity and transcriptomic vulnerability in shaping gray matter atrophy in multiple sclerosis

**DOI:** 10.1126/sciadv.aeg3412

**Published:** 2026-11-11

**Authors:** Mar Barrantes-Cepas, Mario Tranfa, David R. van Nederpelt, Ismail Koubiyr, Luigi Lorenzini, Birgit Helmlinger, Stefan Ropele, Daniela Pinter, Christian Enzinger, Tomas Uher, Manuela Vaneckova, Joep Killestein, Eva M. M. Strijbis, Martijn D. Steenwijk, Hugo Vrenken, Frederik Barkhof, Menno M. Schoonheim, Giuseppe Pontillo

**Affiliations:** ^1^ MS Center Amsterdam, Amsterdam UMC location Vrije Universiteit Amsterdam, Anatomy and Neurosciences, Amsterdam, Netherlands.; ^2^ Amsterdam Neuroscience, Neuroinfection and Neuroinflammation, Brain Imaging, Amsterdam, Netherlands.; ^3^ Department of Radiology and Nuclear Medicine, Amsterdam University Medical Center, Vrije Universiteit, Amsterdam, Netherlands.; ^4^ Department of Advanced Biomedical Sciences, University “Federico II,” Naples, Italy.; ^5^ MS Center Amsterdam, Amsterdam UMC location Vrije Universiteit Amsterdam, Radiology and Nuclear Medicine, Amsterdam, Netherlands.; ^6^ Department of Neuroscience, Rehabilitation, Ophthalmology, Genetics, Maternal and Child Health (DiNOGMI), University of Genoa, Genoa, Italy.; ^7^ Department of Neurology, Research Unit for Neuronal Plasticity and Repair, Medical University of Graz, Graz, Austria.; ^8^ Department of Neurology, Neuroimaging Research Unit, Medical University of Graz, Graz, Austria.; ^9^ Department of Neurology, Medical University of Graz, Graz, Austria.; ^10^ Department of Neurology and Centre of Clinical Neuroscience, First Faculty of Medicine, Charles University in Prague and General University Hospital, Prague, Czechia.; ^11^ Department of Radiology, First Faculty of Medicine, Charles University in Prague and General University Hospital, Prague, Czechia.; ^12^ MS Center Amsterdam, Amsterdam UMC location Vrije Universiteit Amsterdam, Neurology, Amsterdam, Netherlands.; ^13^ Queen Square Institute of Neurology and Centre for Medical Imaging Computing, University College London, London, UK.

## Abstract

Clinical progression is strongly linked to gray matter atrophy in multiple sclerosis (MS), detectable early on MRI and progressing nonrandomly across the brain. However, the mechanisms driving its spatiotemporal progression and individual variability remain unclear. Using MRIs from 2187 participants, alongside normative data, we systematically investigated network-based mechanisms underlying MS-related atrophy. Regional atrophy was associated with functional cortical hubness, supporting the nodal stress hypothesis, and was constrained by anatomical and functional connections, consistent with transneuronal degeneration. Lesional disconnection and transcriptomic vulnerability played marginal roles. Patient- and subgroup-level analyses revealed that network-based mechanisms are specifically linked to MS-related neurodegeneration and may operate differently in distinct subtypes or disease phases. Atrophy patterns were anchored to the connectivity profiles of disease epicenters involving the visual, sensorimotor, and temporal cortices, as well as the hippocampi, amygdalae, and thalami. Network-based measures enhanced the explanation of future atrophy progression in people with MS, providing a mechanistic framework to understand neurodegeneration in MS.

## INTRODUCTION

Clinical progression in multiple sclerosis (MS) is strongly related to gray matter (GM) atrophy, considered as a marker of neurodegeneration and correlating with both physical disability and cognitive impairment (*1*–*3*). The spatiotemporal evolution of regional GM atrophy in MS has been extensively explored: Brain tissue volume loss is present since the very early phases of the disease, progresses with time, and does not occur uniformly throughout the brain, with regions such as the thalamus and the cingulate cortex shrinking faster and earlier than others (*4*). In addition, there is substantial intersubject heterogeneity in MS-related atrophy patterns, which seem to cluster around distinct “modes” of neurodegeneration that do not necessarily align with conventional clinical phenotypes (*5*).

While current evidence clearly highlights that GM atrophy in MS is largely nonrandom (*6*), the mechanisms that drive these spatial and temporal patterns of neurodegeneration remain poorly understood, particularly from an individual-level perspective (*1*, *2*, *7*–*9*), hindering the development of targeted therapeutic strategies aimed at slowing the neurodegenerative component of the disease (*10*). Previous studies have identified where and when MS-related neurodegeneration occurs, focusing primarily on regional characterization and group-level comparisons (*4*, *6*, *9*, *11*). However, they have only provided a limited understanding of the processes underlying GM atrophy and why certain people with MS (pwMS) are more susceptible than others.

Moving beyond regional analyses, a network-based perspective enables the shift in focus to the broader organizational principles of the brain that may underlie vulnerability to disease (*12*–*14*). Recent advances in network neuroscience and transcriptomics, along with the increasing availability of public data resources in these domains, have facilitated the development of a more integrative approach that can now elucidate mechanisms underlying neurodegeneration based on structural and functional connectivity, as well as gene expression profiles from normative data (*15*–*18*). Leveraging this network-based approach, evidence across neurologic and psychiatric disorders suggests that neurodegeneration follows network-constrained patterns (*17*–*19*).

Key mechanisms hypothesized to explain spatiotemporal atrophy patterns include nodal stress, transneuronal degeneration, transcriptomic vulnerability, and lesional disconnection (*1*, *2*, *7*, *8*, *14*, *20*, *21*). Specifically, highly connected regions (i.e., hubs) may be more prone to neurodegeneration, due to their greater metabolic demand leading to cellular dysfunction, a concept known as nodal stress (*1*). In addition, atrophy often mirrors the brain’s intrinsic connectivity, suggesting that damage may spread to functionally and structurally connected regions (i.e., neighbors) via transneuronal degeneration (*3*). Last, this network-based degeneration might be specifically driven by axonal transection through focal lesions, leading to secondary, tract-mediated degeneration of connected GM regions (lesional disconnection) (*14*, *22*). Complementary to these connectivity-based explanations, the concept of transcriptomic vulnerability entails that regions with similar gene expression profiles might have similar biological properties, hence tending to be similarly affected by disease processes (*23*–*25*).

These hypotheses have also been proposed and investigated to some extent in MS (*8*). In particular, GM atrophy has often been correlated with WM disruption (*10*, *20*). Unfortunately, these mechanisms have been studied in isolation, using small, single-site cohorts, with varying imaging modalities and/or analytic methods (*4*, *6*, *9*, *21*). Consequently, their relative contributions to MS-related GM atrophy remain unclear.

Here, we used an integrated approach to systematically investigating the above-mentioned mechanisms in a large multicentric dataset. First, we explored these mechanisms at the group level to characterize robust, “canonical” patterns of MS-related atrophy. Second, we performed subgroup- and individual-level analyses to capture interindividual variability and to determine whether certain patient subgroups may be more susceptible to specific mechanisms. Last, we investigated the potential of such a network-based approach to explaining future atrophy progression.

## RESULTS

### Study population

We retrospectively collected and analyzed structural brain magnetic resonance imaging (MRIs) and clinico-demographic data of pwMS or clinically isolated syndrome (CIS), as well as of healthy controls (HCs), from three European centers, acquired between 2014 and 2023 (Fig. 1). The final study population consisted of 2187 participants (1963 pwMS/CIS, 70% female, 45.3 ± 9.8 years; 224 HCs, 62.5% female, 41.1 ± 12.4 years). For the 565 pwMS/CIS for whom more than one MRI scan was available (total number of sessions = 1245, mean latest follow-up duration = 3.48 years, range: 0.5 to 8.8 years), the first scan was used to model cross-sectional atrophy, while all time points were used to model longitudinal atrophy. Demographic and clinical characteristics of the studied population are given in Table 1, while the different acquisition protocols are detailed in table S1, and further information regarding image quality control and excluded participants is provided in fig. S1 and table S2.

**Fig. 1.**
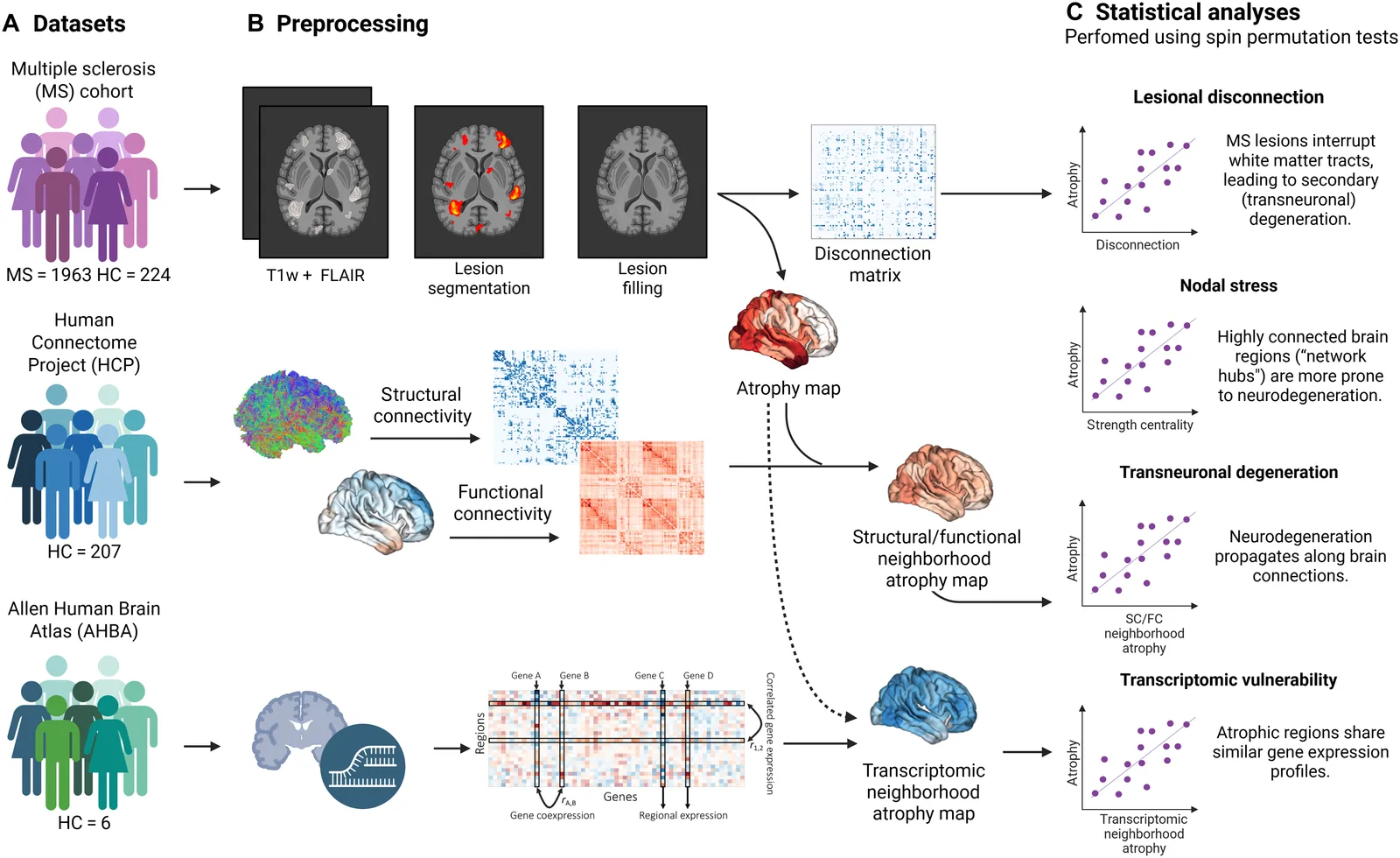
Study workflow. (**A**) The study integrated three datasets: MS cohort and HCs, including T1w and FLAIR scans; the HCP, providing structural and functional connectomes (SC/FC); and the AHBA, providing gene expression data. (**B**) In the MS cohort, lesions were segmented using LST-AI, and subsequently, T1w scans were filled with NiftySeg. Disconnectome matrices were computed using the LQT, while cortical thickness and subcortical volumes were extracted with FreeSurfer. Atrophy maps were generated using Cohen’s *d*, and connectome and gene expression data were preprocessed using the ENIGMA toolbox. Neighboring atrophy maps were obtained as described in Materials and Methods (*18*). (**C**) Statistical analyses included spin permutation tests (*n* = 10,000) to assess spatial correspondence. (B) Reprinted from NeuroImage, vol. 244, Arnatkeviciute A., Fulcher B. D., Bellgrove M. A., Fornito A., “Where the genome meets the connectome: Understanding how genes shape human brain connectivity,” Article 118570, Copyright (2021), with permission from Elsevier, as well as from NeuroImage: Clinical, vol. 40, Vo A., Tremblay C., Rahayel S., Shafiei G., Hansen J.Y., Yau Y., Mišić B., Dagher A., “Network connectivity and local transcriptomic vulnerability underpin cortical atrophy progression in Parkinson’s disease,” Article 103523, Copyright (2023), with permission from Elsevier. [(A) to (C)] Created with BioRender. Schoonheim, M. (2026) https://BioRender.com/nf6xdo8.

**Table 1.** Demographic, clinical, and MRI characteristics of the studied population at baseline. BPF, brain parenchymal fraction; DMT, disease-modifying treatment; EDSS, Expanded Disability Status Scale; IQR, interquartile range; PPMS, primary-progressive MS; SPMS, secondary-progressive MS; TLV, total lesion volume.

	HC	pwMS/CIS	*P* value
*N*	224	1963	
Age [years], mean± SD	41.1 ± 12.4	45.3 ± 9.84	<0.001*
Female, *n* (%)	140 (62.5%)	1375 (70%)	0.02†
Disease characteristics			
Phenotype, *n* (%)			
CIS		57 (2.9%)	–
RRMS		1620 (82.5%)	–
SPMS		242 (12.3%)	–
PPMS		44 (2.2%)	–
Disease duration [years], mean± SD		14.5 ± 9.2	–
EDSS, median (range)		2.5 [0–8.5]	–
DMT, *n* (%): yes/no		1505 (76.7%) / 458 (23.3%)	–
TLV [ml], median (IQR)		3.36 [1.07–9.24]	–
BPF, mean ± SD	0.77 ± 0.02	0.75 ± 0.05	<0.001*

* Permutation *t* test (*n* = 10,000).

† Chi-square test.

### GM atrophy in MS

We processed three-dimensional (3D) T1-weighted (T1w) and 3D fluid-attenuated inversion recovery (FLAIR) scans to obtain values of thickness from 100 cortical regions [Schaefer atlas (*26*)] and volume from 14 subcortical structures [FreeSurfer’s Aseg atlas (*27*)], including the hippocampus. Although phylogenetically classified as allocortex, the hippocampus is operationally processed using volume-based segmentation pipelines and conventionally grouped with subcortical structures in neuroimaging studies. Therefore, while we refrain from directly labeling the hippocampus as subcortical, it is included whenever we discuss subcortical structures as a collective group throughout the manuscript. Individual regional atrophy was expressed as *W*-scores, reflecting the deviation from the HC group while adjusting for the physiological effects of age, sex, intracranial volume (for subcortical volumes), and site. When comparing *W*-score maps (sign-flipped so that more positive scores indicated greater atrophy), pwMS/CIS exhibited widespread reduction of subcortical GM volumes relative to HCs, most pronounced in the thalami (Cohen’s *d* = 1.07, *P*_FDR_ < 0.001). Significant cortical thinning was evident in the temporo-polar, sensorimotor, posterior cingulate, and precuneal cortices (Fig. 2).

**Fig. 2.**
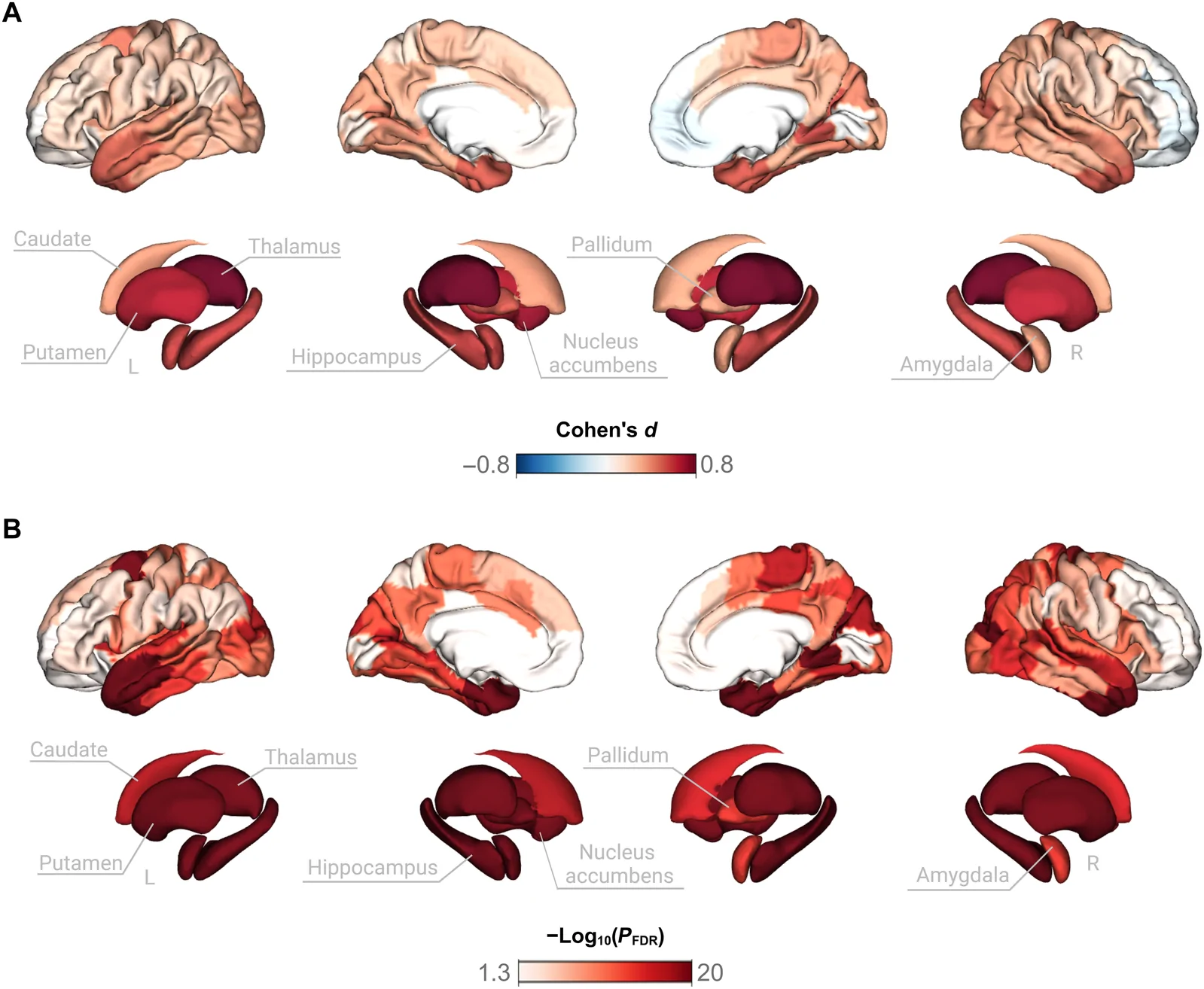
GM atrophy in pwMS/CIS. (**A**) Atrophy maps show cross-sectional differences (Cohen’s *d*) in cortical thickness and subcortical volumes between pwMS/CIS and HCs, adjusting for physiological effects of age, sex, intracranial volume (for subcortical volumes), and site. (**B**) Negative log_10_-transformed FDR-corrected *P* values are shown.

### Network mechanisms shape GM atrophy

We operationalized the nodal stress, transneuronal degeneration, lesional disconnection, and transcriptomic vulnerability hypotheses in terms of spatial similarity between regional atrophy and a set of network-based maps. Statistical significance of spatial correlations was assessed against null distributions obtained by spatially permuting maps randomly (10,000 repetitions), with significance set at *P* < 0.05 (*16*).

To investigate the nodal stress hypothesis (i.e., highly connected regions are more prone to neurodegeneration), we investigated the association between regional GM atrophy and functional and structural hubness maps, obtained using normative data derived from 207 healthy adults sampled from the Human Connectome Project (HCP), and expressed in terms of strength centrality (i.e., the sum of all cortico-cortical or subcortico-cortical edges insisting on each node) (Fig. 3, A and B). We found a positive correlation between functional cortico-cortical strength centrality and cortical atrophy (*r* = 0.38, *P* = 0.01; Fig. 3C), indicating that cortical regions with stronger functional hub characteristics tended to exhibit greater atrophy. In contrast, no associations with regional atrophy were found for functional subcortico-cortical strength centrality (*r* = 0.31, *P* = 0.15; Fig. 3C), structural cortico-cortical strength centrality (*r* = −0.15, *P* = 0.21; Fig. 3D), or structural subcortico-cortical strength centrality (*r* = 0.35, *P* = 0.11; Fig. 3D).

**Fig. 3.**
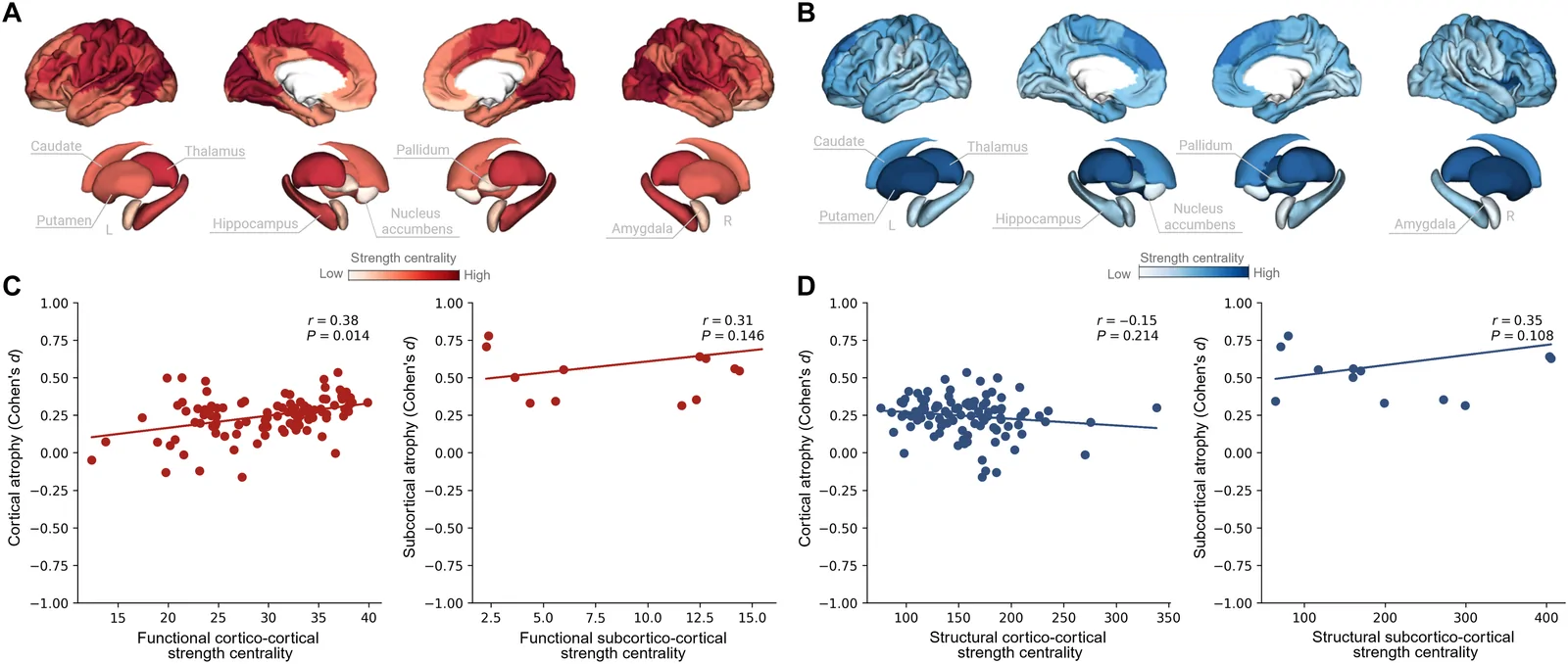
Functional cortical hubs exhibit greater GM atrophy in pwMS/CIS. (**A**) Functional and (**B**) structural strength centrality maps obtained using the HCP data. (**C**) Spatial correlation between functional and (**D**) structural strength centrality and GM atrophy.

For transneuronal degeneration (i.e., neurodegeneration propagates along brain connections), we tested the association between regional GM atrophy and neighborhood atrophy maps, defined as the average atrophy of each region’s first-degree neighbors, weighted by the strength of their functional or structural connections (Fig. 4, A and B) (*18*). We found a positive correlation between cortical atrophy and both functional (*r* = 0.41, *P* = 0.005; Fig. 4C) and structural (*r* = 0.51, *P* = 0.004; Fig. 4D) cortico-cortical neighborhood atrophy, indicating that cortical regions tend to lose volume together with their functionally and structurally connected neighbors. No associations with atrophy were observed for subcortical regions and functional (*r* = 0.29, *P* = 0.16; Fig. 4C) or structural (*r* = 0.13, *P* = 0.35; Fig. 4D) neighborhood atrophy. To verify that our transneuronal degeneration analyses were not merely capturing underlying spatial proximity gradients, we conducted additional partial spatial permutation tests. Specifically, we tested the association between regional GM atrophy and connectivity-weighted neighborhood atrophy maps while adjusting for a neighborhood atrophy map weighted by physical proximity, computed as the reciprocal of the Euclidean distance between pairs of regions. The associations between cortical atrophy and both functional (*r* = 0.31, *P* = 0.023) and structural (*r* = 0.44, *P* = 0.014) cortico-cortical neighborhood atrophy remained significant, highlighting the unique role of connectivity-constrained neurodegeneration beyond anatomical proximity.

**Fig. 4.**
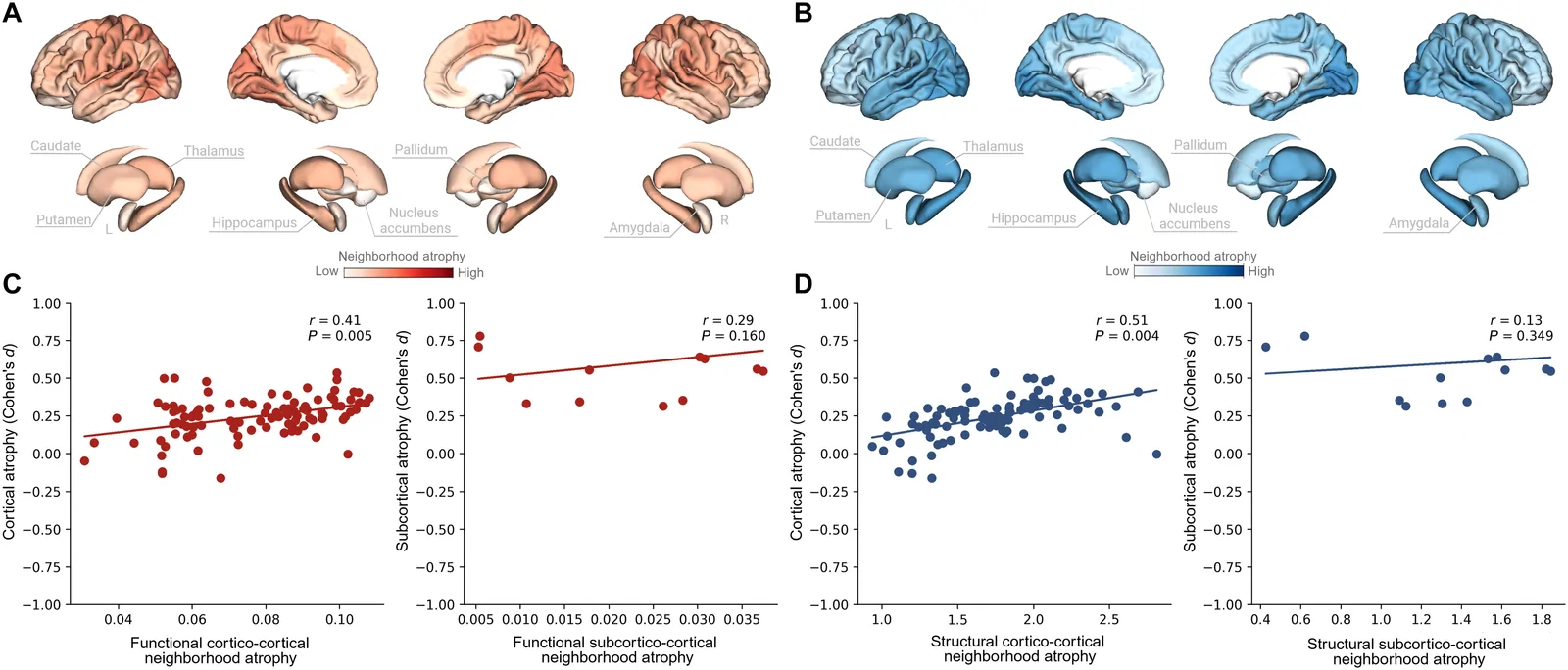
Connected cortical regions tend to atrophy together in pwMS/CIS. (**A**) Functional and (**B**) structural neighborhood atrophy maps, defined as the average atrophy of each region’s first-degree neighbors, weighted by connection strength. (**C**) Spatial correlation between functional and (**D**) structural neighborhood atrophy and GM atrophy.

We then investigated whether atrophy overlapped with lesional disconnection (i.e., WM lesions drive secondary neurodegeneration in anatomically connected regions). We obtained data-driven structural disconnectomes by overlaying individual lesion masks in Montreal Neurological Institute (MNI) space onto the HCP1065 normative tractography atlas using the Lesion Quantification Toolkit (LQT) (*28*). This approach provides a tractography-informed estimate of how focal WM lesions may disrupt large-scale structural connectivity, measuring the pairwise disconnection between pairs of GM regions as the proportion of connecting streamlines intersecting lesions. PwMS displayed the highest lesion probability in the periventricular WM, with the highest disconnection probability at the level of the occipital WM, commissural fibers, and long-range frontal and temporal association tracts (fig. S2). Then, we tested the spatial correlation between regional GM atrophy and node disconnection, expressed as the regional strength centrality of the disconnectome matrix (i.e., the sum of all cortico-cortical or subcortico-cortical edges insisting on each node) (Fig. 5A). On average, lesion-related GM disconnection was more prominent in the deep GM and in temporal and posterior cortical regions (Fig. 5A). We found no relationship between cortical GM atrophy and structural disconnection (*r* = 0.16, *P* = 0.10; Fig. 5C). Similarly, no relationship was observed between subcortical atrophy and structural disconnection (*r* = 0.10, *P* = 0.35; Fig. 5C).

**Fig. 5.**
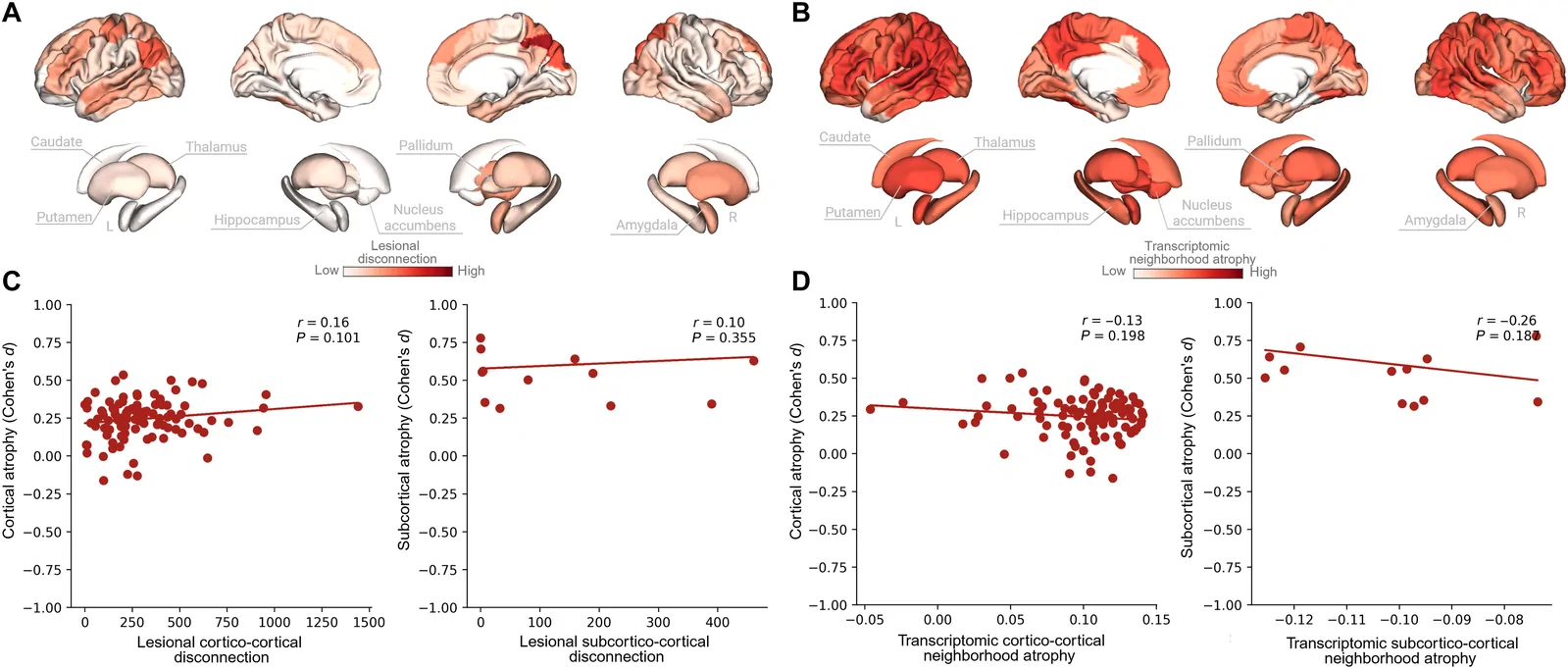
No associations between GM atrophy and lesional disconnection or transcriptomic vulnerability in pwMS/CIS. (**A**) MS lesion disconnection maps obtained with a normative tractography atlas. (**B**) Transcriptomic neighborhood atrophy, defined as the average atrophy of each region’s first-degree neighbors, weighted by transcriptomic similarity. (**C**) Correlation of lesion-related disconnection and (**D**) transcriptomic neighborhood atrophy and GM atrophy.

Last, for transcriptomic vulnerability (i.e., regional neurodegeneration is mediated by shared gene expression profiles), we tested the association between regional GM atrophy and transcriptomic neighborhood atrophy maps. The latter was defined as the average atrophy of each region’s first-degree neighbors, weighted by their normative transcriptomic similarity, derived from the Allen Human Brain Atlas (AHBA) (Fig. 5B). In the cortex, no relationship was found (*r* = −0.13, *P* = 0.2; Fig. 5D), as well as in subcortical regions (*r* = −0.26, *P* = 0.19; Fig. 5D).

### Patient- and subgroup-tailored atrophy modeling

We adapted our network-based models to assess whether they configured atrophy patterns in individual pwMS/CIS and specific subgroups. Specifically, we assessed the spatial correlations between individual atrophy (i.e., *W*-score) maps and network-based maps on a per-patient basis and Fisher’s *z*-transformed the obtained *r* values to enable comparisons across individuals and subgroups.

At the individual level, all observed associations were significantly different from zero (*P*_FDR_ < 0.05 at one-sample *t* tests), indicating relatively consistent effects across pwMS/CIS (Fig. 6A). The greatest effects were observed for the association between regional GM atrophy and both structural (Cohen’s *d* = 1.76) and functional (*d* = 0.61) cortico-cortical neighborhood atrophy, supporting the role of transneuronal degeneration as the main mechanism shaping MS-related atrophy patterns. We also assessed the proportion of participants showing significant associations with regional GM atrophy for each mechanism at individual spatial permutation tests, which was the highest for structural and functional cortico-cortical neighborhood atrophy (50.8 and 28.5%, respectively) (Fig. 6B). Associations between subcortical atrophy and network-based maps exhibited the greatest variability across pwMS/CIS, suggesting higher heterogeneity in the mechanisms driving deep GM neurodegeneration.

**Fig. 6.**
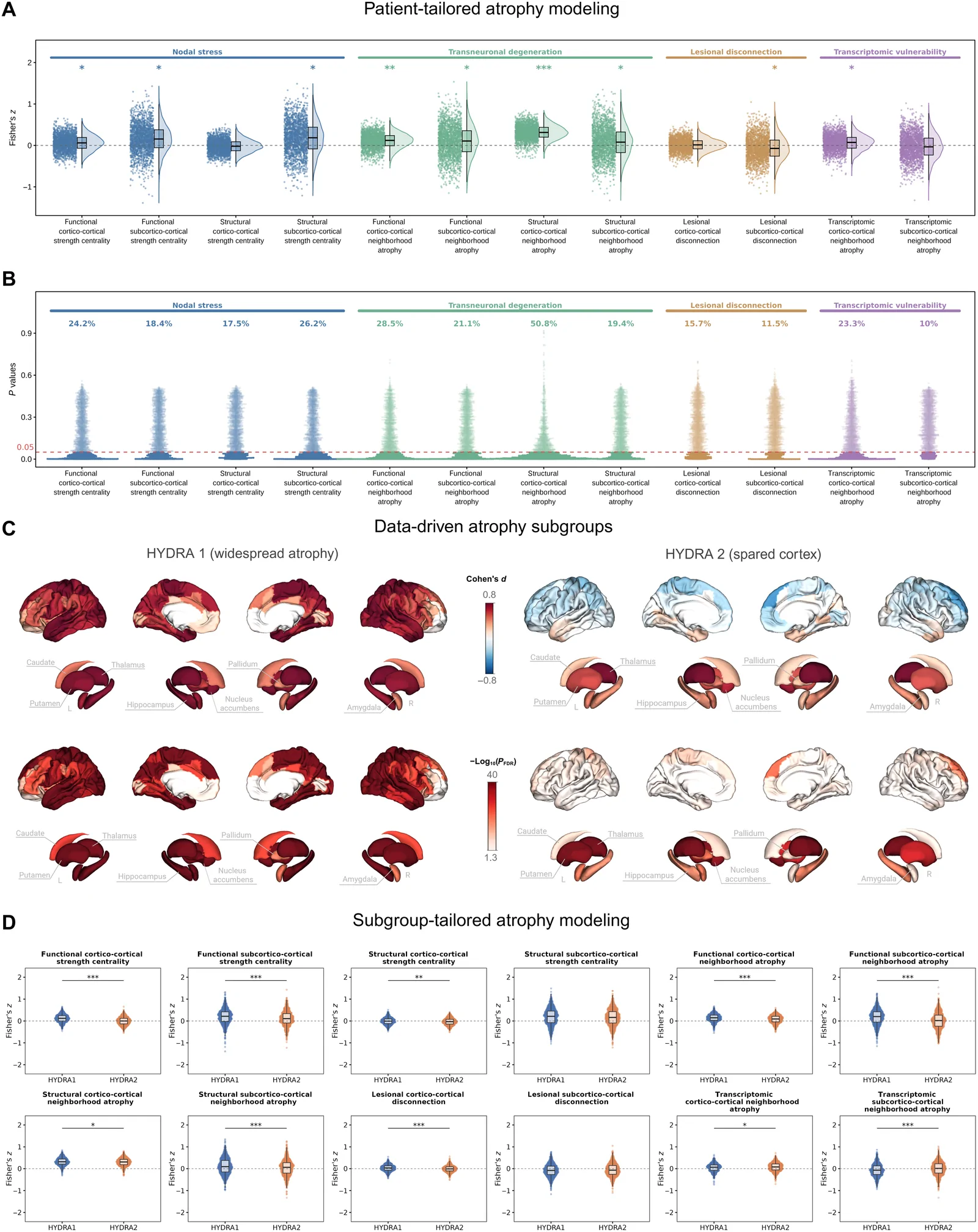
Patient- and subgroup-tailored atrophy modeling*.* (**A**) Fisher *z*-transformed individual correlation coefficients are shown for each mechanism. All mechanisms were significant against the null using one sample *t* test and false discovery rate (FDR)–corrected *P* values. Effect sizes are indicated as: * = small Cohen’s *d* (0.2 to 0.5), ** = moderate Cohen’s *d* (0.5 to 0.8), *** = large Cohen’s *d* (≥0.8). (**B**) *P* values for each mechanism at the individual level, with the percentage of significant cases shown above each distribution. (**C**) Atrophy maps showing effect sizes (Cohen’s *d*) and negative log_10_-transformed FDR-corrected *P* values for the data-driven HYDRA groups. (**D**) Boxplots comparing HYDRA subgroups across different mechanisms. *P < 0.05, **P < 0.01, and ***P < 0.001.

We then investigated the role of the different network-based mechanisms in shaping GM atrophy in distinct pwMS/CIS subgroups. First, we used the HYDRA algorithm, a nonlinear semisupervised machine learning approach, to subgroup patients into distinct regional atrophy profiles (*29*). We identified two main GM atrophy phenotypes: The first subgroup exhibited widespread cortical and subcortical atrophy, while the second showed selective subcortical GM volume loss, with relatively preserved cortical thickness (Fig. 6B and fig. S3). PwMS/CIS assigned to the two clusters were slightly different in terms age (*P* < 0.001), sex (*P* < 0.001), lesion volume (*P* < 0.001), and clinical phenotype (*P* = 0.016) but did not differ in disease duration (DD; *P* = 0.69) or physical disability level (*P* = 0.19) (table S3). When investigating neurodegeneration mechanisms in the two data-driven subgroups, we observed that network-based mechanisms were consistently more relevant in the widespread atrophy subgroup than in individuals with spared cortical GM (Fig. 6C). Similarly, we compared neurodegeneration mechanisms across clinical phenotypes, defined as early relapsing [ER: CIS + relapsing-remitting MS (RRMS) with a DD below 5 years, *N* = 360], late relapsing (LR: RRMS with a DD above or equal to 5 years, *N* = 1317), secondary progressive (SP, *N* = 242), and primary progressive (PP, *N* = 44) (*30*). We observed that associations between GM atrophy and network-based maps (especially those indexing nodal stress and transneuronal degeneration) were stronger in more advanced (LR and SP) compared to earlier (ER) phases of the disease, with PP patients showing intermediate behavior (fig. S4).

### Data-driven disease epicenters

We investigated whether MS-related atrophy patterns were anchored to the connectivity profiles of specific regions. To identify these connectivity-based disease “epicenters” (i.e., critical regions where the disease process supposedly begins to then spread “transneuronally” to other brain regions facilitated by the underlying connectivity patterns), we used a previously adopted approach (*31*). Briefly, we correlated the structural and functional connectivity profiles of each brain region with individual atrophy maps and repeated this systematically across all regions. The resulting correlation coefficients were *r*-to-*z* transformed, and one-sample, one-tailed *t* tests were performed for each region to assess their significance at the group level. Disease epicenters thus represented regions whose functional and structural connectivity profile spatially resembled MS-related atrophy maps.

Both structural and functional analyses revealed overlapping candidate epicenter regions within the visual and temporal cortices (Fig. 7). Cortical functional epicenters also extended to the sensorimotor cortex. In addition, the thalamus, hippocampus, and amygdala were identified as structural epicenters of neurodegeneration, with the latter two also emerging as functional epicenters.

**Fig. 7.**
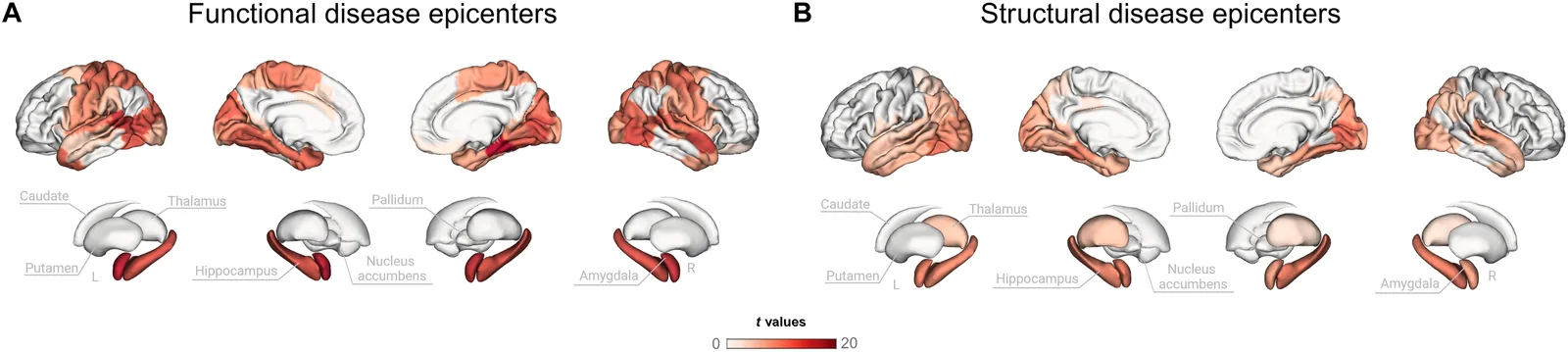
Distribution of structural and functional disease epicenters. Individual-level (**A**) functional and (**B**) structural disease epicenters were computed, Fisher *r*-to-*z* transformed, and assessed for group-level significance using one-tailed *t* tests with FDR correction. Regions exhibiting significant effects were delineated from nonsignificant ones. The map displays corresponding *t* values.

### Findings were robust across different parcellation schemes

In sensitivity analyses using (i) a more fine-grained subdivision of the cortex based on the 400-parcel version of the Schaefer atlas and (ii) the Glasser HCP multimodal atlas, dividing the cortex into 360 parcels based on a combination of structural and functional features (*32*), we showed that our findings were substantially robust across different parcellation resolutions and strategies (figs. S5 to S8).

### Sensitivity analysis using a different subtyping and staging algorithm

As a further sensitivity analysis, we examined whether the HYDRA subtypes and the identified disease epicenters were reproducible using an alternative subtyping and staging method. We applied Subtype and Stage Inference (SuStaIn), an unsupervised machine learning algorithm that combines concepts from clustering and event-based modeling to infer data-driven subgroups characterized by common patterns of disease progression (subtypes) and abnormality levels (stages) (*33*). We identified two subtypes, a subcortex-first subtype and a cortex-first subtype, which substantially aligned with HYDRA clusters (the subcortex-first subtype with HYDRA’s spared cortex cluster and the cortex-first with HYDRA’s widespread atrophy, respectively) and replicated subgroup-level associations (figs. S9 and S10 and table S4). When looking at the inferred sequences of biomarker abnormalities, atrophy of the thalamus, as well as of the visual and sensorimotor cortices, was an early event across subtypes, indirectly partially confirming the epicenter mapping results.

### A network-based model explains longitudinal atrophy in individual patients

Last, we evaluated whether the investigated mechanisms could explain future progression of regional brain atrophy. We modeled longitudinal atrophy progression in each brain region at the group level using linear mixed-effects models with time points nested within pwMS/CIS. At the patient level, region-wise estimates of longitudinal atrophy progression were expressed in terms of annualized percentage change (sign-flipped so that more positive values indicated greater atrophy progression) obtained from individual linear models. Patients showed widespread volume/thickness loss over time, more prominent in the temporal, cingulate, and sensorimotor cortices and in subcortical structures (fig. S11).

We specified generalized additive models (GAMs) using baseline information to explain subsequent atrophy progression in individual patients. We combined data from all pwMS/CIS and all regions to fit a single unified model including nonlinear terms for network-based measures (i.e., normative structural strength centrality, normative functional strength centrality, baseline lesional disconnection, and functional, structural, and transcriptomic neighborhood atrophy), as well as baseline atrophy, age, follow-up duration, spatial autocorrelation (based on each region’s center of gravity coordinates), random intercepts for patients and region, and a random slope for each region’s relationship between baseline atrophy and subsequent atrophy. The linear fixed effects included sex, site, and a binary variable indicating whether each region was cortical or subcortical. For comparison, we also built a baseline model only including nonnetwork-based variables.

The network-based model explained 30.3% deviance (adjusted *R^2^* = 0.30) in longitudinal regional atrophy progression, with significant effects of almost all network-based measures. A complete list of explanatory variables, along with their significance and the corresponding interpretation, is provided in Table 2, while a graphical depiction of the model terms is available in fig. S12. Compared to the baseline model not including network measures, the addition of baseline network-based measures significantly enhanced the explanatory power toward subsequent atrophy progression [Akaike information criterion (AIC): 518,785.0 versus 519,108.9, *P* < 001 at the likelihood ratio test].

**Table 2.** GAM for explaining longitudinal atrophy progression. Significance and interpretation of the effect of each explanatory variable on the annualized percentage rate of subsequent longitudinal atrophy.

Explanatory variables	*t*/*F* statistic	*P* value	Interpretation
Global intercept	3.36 (*t*)	< 0.001	Average longitudinal atrophy
Male sex	1.88 (*t*)	0.06	Not significant
Cortical/subcortical node	6.08 (*t*)	< 0.001	Greater longitudinal atrophy in subcortical nodes
Age	745.433 (*F*)	0.12	Not significant
Baseline atrophy	1402.91 (*F*)	< 0.001	Longitudinal atrophy decreases for increasing baseline atrophy, especially for extreme values (ceiling effect)
Subject intercept	94.00 (*F*)	< 0.001	Different subjects have different longitudinal atrophy
Node intercept	7.98 (*F*)	< 0.001	Different nodes exhibit different longitudinal atrophy
Node random slope	12.55 (*F*)	< 0.001	Different nodes have different relationships between baseline and longitudinal atrophy
Spatial autocorrelation	26.44 (*F*)	< 0.001	Close nodes have more similar longitudinal atrophy
Structural strength centrality	20.79 (*F*)	0.04	Intermediate values associated with the lowest longitudinal atrophy
Functional strength centrality	0.00 (*F*)	0.64	Not significant
Lesional disconnection	68.89 (*F*)	< 0.01	Greater disconnection is associated with greater longitudinal atrophy, with possible ceiling effect
Structural neighborhood atrophy	59.80 (*F*)	< 0.001	Extremely high values associated with greater longitudinal atrophy
Functional neighborhood atrophy	80.34 (*F*)	< 0.001	Intermediate values associated with the greatest longitudinal atrophy
Transcriptomic neighborhood atrophy	43.79 (*F*)	< 0.001	Extremely high values associated with greater longitudinal atrophy

## DISCUSSION

Using structural MRI from a large multicentric cohort and a wide set of brain network analyses, we systematically investigated specific hypothesis-driven network-based mechanisms driving GM atrophy in pwMS/CIS. We showed that MS-related atrophy was associated with functional cortical hubness, supporting the nodal stress hypothesis. In addition, transneuronal degeneration emerged as a relevant mechanism of neurodegeneration, suggesting that MS-related atrophy is constrained by anatomical and functional connections. A less important role was observed for lesional disconnection and transcriptomic vulnerability. These network-based mechanisms appeared to be specifically linked to MS-related neurodegeneration rather than to the brain’s physiological structural organization and may operate differently in distinct subgroups of pwMS/CIS or phases of the disease. Neurodegeneration patterns are anchored to the connectivity profiles of distinct regions, pointing to the visual, sensorimotor, and temporal cortices, as well as to the thalamus, hippocampus, and amygdala, as putative disease epicenters. Last, we showed that network-based measures, which are easily obtainable from conventional MRI and normative datasets, could be used to explain future atrophy progression in individual patients.

Functional cortical hubs were associated with greater atrophy, supporting the nodal stress hypothesis (*19*, *34*). Although the effect size was relatively modest, these findings are consistent with prior research showing that functional hubness is associated with disease vulnerability across conditions, including MS (*16*, *35*–*37*). Regions with high functional centrality, subject to heavy network traffic, may undergo activity-related “wear and tear” that gives rise to or worsens disease-related neurodegeneration, possibly due to high metabolic demands (*35*). In contrast, structural hubs did not show associations with atrophy, which might suggest that highly anatomically connected cortical regions, essential for the integration of brain functioning, are more protected against degeneration through compensatory mechanisms. We also observed that the regional cortical atrophy level was higher for strongly anatomically and functionally connected regions, even when adjusting for anatomical proximity. This finding supports the transneuronal degeneration hypothesis, suggesting that neurodegeneration may propagate along brain connections via anterograde and/or retrograde trans-synaptic damage. Such network-constrained patterns are increasingly recognized in primary neurodegenerative disorders, where pathological changes are thought to spread from regions of greatest/earliest vulnerability (i.e., disease epicenters) to connected areas (*18*, *38*, *39*). In this context, network architecture has been hypothesized to provide “passive” anatomical conduits through which pathological agents (e.g., misfolded proteins) can be transported (the so-called prion-like hypothesis), as well as to support a more “active” link between altered dynamics of neuronal circuits and neurodegeneration (*38*, *40*). Similar mechanisms might also play a role in MS, where the propagation of neuroinflammatory response along WM tracts has also been hypothesized (*41*).

For lesional disconnection and transcriptomic vulnerability, no associations with regional GM atrophy were observed. Previous studies have investigated the relationship between lesions and atrophy in MS, showing that disruption of WM integrity can lead to trans-synaptic degeneration and, therefore, cortical thinning and subcortical GM volume loss in connected regions (*42*). Similar patterns of secondary GM degeneration following WM damage have also been observed in other neurological disorders, such as stroke and traumatic brain injury disease (*43*, *44*). One potential explanation for the apparently discrepant results observed in our study is that the impact of lesion-induced disconnection on connected GM regions may peak relatively early after the occurrence of a new lesion, to eventually reach a plateau and be superseded by other neurodegeneration mechanisms (*42*). According to this interpretation, the snapshot provided by cross-sectional analyses might not be sensitive enough to detect such an effect, which would be fully exposed only with high-frequency serial acquisition capturing lesion dynamics and subsequent changes in connected GM regions. Also, it should be highlighted that binary lesion masks do not capture lesion heterogeneity and that macroscopic lesions only represent the tip of the iceberg of WM damage in MS. More advanced techniques (e.g., diffusion or quantitative MRI) assessing brain tissue microstructure potentially could provide a more precise account of WM integrity and, consequently, of its impact on regional GM atrophy (*45*, *46*). As for the lack of association between normative gene expression profiles and regional GM atrophy, this falls in line with the knowledge of MS as primarily linked to a dysfunction of the immune system rather than to the selective vulnerability of a specific cell population in the central nervous system. However, it should be noted that the normative data are based on a limited number of cases and do not include possible MS-specific alterations in transcriptomic profiles. Hence, future studies focusing on specific genes and/or relying on spatial transcriptomics on MS brain tissues (*25*) will be needed to unravel the spatiotemporal and cell type–specific gene expression profiles associated with neurodegeneration.

Although the associations between network-based maps and regional atrophy did not reach statistical significance for subcortical regions, they showed similar trends to those observed for cortical areas. This may reflect the fact that subcortical structures exhibit substantial atrophy, potentially diminishing the detectability of network-driven effects, possibly reflecting a floor effect. In addition, the smaller number of subcortical regions compared to cortical regions reduces the statistical power, further limiting the likelihood of significant associations. Moreover, segmentation of subcortical GM using FreeSurfer is known to be less reliable than for cortical GM (*47*), especially in pwMS (*48*), and alternative tools may provide more accurate measures in the future.

Overall, these findings suggest that network-based mechanisms shape MS-related neurodegeneration patterns. Traditionally, MS pathology has been primarily attributed to focal lesions and (acute) inflammation. While current therapies effectively target (acute) inflammatory activity, it is now clear that disease progression can occur independently of relapses and acute inflammation. We speculate that nodal stress and transneuronal degeneration might play an important role in driving neurodegeneration regardless of lesions, potentially representing treatment targets to tackle progression independent of new lesion activity (*49*, *50*).

We then tailored our analyses to individual patients, aiming to assess whether distinct mechanisms affect some pwMS more than others. While the average trends of individual-level results broadly recapitulated the findings of the group-level analyses (i.e., the mechanisms showing stronger effects at the group level also tended to exhibit higher effect sizes at the individual level), with regional GM atrophy most strongly associated with that of connected neighbors, individual-level analyses revealed a specific degree of intersubject variability which was not discernible at the group level, especially for subcortical regions. This may reflect greater biological heterogeneity in the mechanisms driving subcortical neurodegeneration in MS, although methodological reasons such as the greater measurement error associated with smaller brain structures (*47*, *51*) plus the lower stability of correlations drawn from a smaller number of regions might also play a role.

When looking at data-driven atrophy subgroups, we identified two distinct patterns: one characterized by widespread subcortical and cortical neurodegeneration and the other showing predominantly subcortical atrophy with relative sparing of the cortex. Patients assigned to the two subgroups exhibited similar DDs, as well as comparable proportions of clinical phenotypes, suggesting that the observed GM atrophy profiles do not represent consecutive phases of the disease but rather two distinct modes of neurodegeneration, which substantially align with the results of our sensitivity analyses using a different subtyping and staging algorithm and replicating previously described MRI-driven subtypes (*5*, *52*). When comparing hypothesized neurodegeneration drivers across MRI-driven and clinical phenotypes, significant differences emerged, with network-based mechanisms apparently more relevant in patients with widespread atrophy and more advanced disease phases. Together, results of the subgroup-level analyses seem to suggest that the hypothesized network-based mechanisms are specifically associated with MS-related neurodegeneration, rather than reflect the physiological organization of brain structure (i.e., their associations with regional volume/thickness only become apparent in the presence of disease-related atrophy), and may have varying impact according to the phase and phenotype of the disease.

We also tested the hypothesis that the connectivity profiles of specific regions constrain the observed atrophy patterns. We identified candidate structural and functional epicenters in both cortical and subcortical regions, from which the propagation of neurodegeneration to connected regions supposedly starts. Cortical epicenters were primarily located in the visual and temporal areas, as well as in the sensorimotor cortex, possibly reflecting regions of high connectivity that may facilitate network-level spread of atrophy. Additional epicenter regions included the hippocampus, amygdala, and thalamus. These findings can be interpreted in light of existing knowledge on MS. For instance, involvement of optic pathways, including secondary atrophy of the visual cortex, is a common early manifestation of the disease (*53*), as well as thalamic atrophy (*54*). It is important to note that our epicenter mapping analysis mostly assumes transneuronal degeneration as the mechanism driving neurodegeneration, which might not be fully accurate. While the observed epicenters provide a meaningful approximation, more complex models taking into account other mechanisms and/or incorporating longitudinal assessments are warranted to fully understand how neurodegeneration propagates across the brain. Our core results were substantially confirmed across a range of sensitivity analyses adopting alternative parcellation schemes or subtyping and staging algorithms, suggesting that the findings remain relatively robust and independent of contingent methodological choices.

Longitudinal modeling showed that network-derived metrics significantly explain variance in atrophy progression that is not accounted for by baseline damage and demographic factors. Baseline atrophy was a significant explanatory variable of subsequent atrophy progression, which decreased for increasing baseline atrophy, especially for extreme values, possibly reflecting a ceiling effect of structural brain damage. Along with spatial autocorrelation (i.e., close regions have more similar longitudinal atrophy) and subcortical vulnerability (i.e., subcortical regions exhibit greater longitudinal atrophy), all network-based regional properties, except for functional network centrality, emerged as significant explanatory variables of longitudinal atrophy. Notably, lesional disconnection and neighborhood atrophy measures, which are (partially) derived from patient-level data, were more significantly associated with longitudinal atrophy progression than network centrality measures, which are purely based on normative connectomes. This suggests that patient-specific network information might be more strongly linked to longitudinal atrophy progression, warranting future studies using advanced MRI to obtain individual-level structural and functional connectomes (*55*).

Several additional limitations should be acknowledged. First, although our cohort included data from multiple European centers, DD was relatively long on average, meaning that the earliest stages of MS were underrepresented. Second, we analyzed cortical and subcortical regions separately to mitigate the confounding effects of spatial autocorrelation, but this choice limited our ability to fully characterize the entire brain network and subcortico-subcortical interactions, which may also play an important role in disease propagation. Third, the mostly cross-sectional nature of our study does not allow for strong causality implications regarding neurodegeneration mechanisms. Also, because our spatial similarity analyses serve as indirect proxies rather than direct measurements of biological processes, future studies using longitudinal designs and more biologically specific measurements (e.g., from diffusion or quantitative MRI) are warranted to establish causality and disentangle these competing mechanisms. Last, we used normative connectomes rather than patient-level structural and functional networks. For cross-sectional analyses, our objective was to capture a reference (i.e., premorbid) architecture of brain connectivity, to assess how it constrains disease-related neurodegeneration, rather than characterizing MS-induced connectivity alterations. In this regard, the HCP dataset provides a robust estimate of typical brain network organization before MS-related changes, especially since the mean age of the HCP cohort (∼29 years) aligns with the typical age of MS onset (20 to 40 years). We do, however, acknowledge that using normative data serves as a proxy; ideally, direct assessment would use predisease longitudinal data, although such data are almost impossible to have in MS cohorts. As for the longitudinal analysis seeking to explain future atrophy progression, only approximately 100 patients in our dataset had complete baseline diffusion and functional MRI acquisitions and longitudinal MRI visits, some of whom had relatively short follow-up durations (<2 years). Given these limitations, this subset would have been insufficiently powered for building robust GAMs. Future longitudinal studies should aim to incorporate actual data from individual pwMS to better reflect disease-specific characteristics and their heterogeneity across participants.

In conclusion, MS-related GM atrophy is shaped by network architecture, with nodal stress and transneuronal degeneration emerging as possible mechanisms of neurodegeneration. Visual, sensorimotor, and temporal cortices, as well as the hippocampi and thalami, may act as epicenters of neurodegeneration. Network-based measures, which are easily obtainable from conventional MRI and normative datasets, can help explain future atrophy progression in individual patients. These findings provide a mechanistic framework for understanding MS-related neurodegeneration, potentially informing patient prognostication and therapeutic approaches.

## MATERIALS AND METHODS

Unless otherwise specified, statistical analyses were performed in R (v4.4.1, R Foundation).

### Participants

In this retrospective multicentric study, we collected structural brain MRI and clinico-demographic data of patients diagnosed with MS according to the 2017 McDonald criteria (*56*) or CIS (*57*), as well as of HCs. Included participants were part of the Amsterdam, Prague, and Graz MS cohorts (*58*, *59*). Exclusion criteria were age younger than 18 or older than 75 years, the presence of other relevant neurologic, psychiatric, or systemic conditions, and having had relapses or received steroid treatment within the month preceding the visit. Written informed consent was obtained from each participant independently at each center following standard procedures. The local ethical committee approved the study protocol, and written informed consent was obtained from all individuals according to the Declaration of Helsinki (NL74887.029.20).

### MRI acquisition and processing

Participants underwent longitudinal brain MRI on 3T scanners including 3D T1w and 3D FLAIR sequences (table S1). T2-hyperintense lesions were automatically segmented on FLAIR and T1w scans using LST-AI (*60*), and the corresponding masks were used to fill lesions in T1w images through NiftySeg’s lesion filling procedure (*61*).

Lesion-filled T1w volumes were processed with FreeSurfer (v7.4.1) using the recon-all cross-sectional and longitudinal pipelines to obtain thicknesses of 100 cortical regions from the Schaefer atlas (*26*) and volumes of 14 subcortical structures from FreeSurfer’s Aseg atlas (*27*). To ensure high quality of the included scans, we used the Euler number, an index of cortical surface reconstruction quality generated automatically by FreeSurfer. In particular, we discarded scans below an Euler number cutoff threshold of −120 (*62*).

In addition, disconnection matrices were estimated from lesion masks and the HCP1065 tractography atlas using LQT (*28*). Briefly, individual lesion masks were registered to the MNI space by applying the nonlinear transformation obtained by normalizing T1w volumes to the template using ANTs v2.5.1. Then, on the basis of spatially normalized lesion masks, the pairwise disconnection between pairs of GM regions was computed as the proportion of streamlines intersecting lesions and used to fill subject-level 114 × 114 structural disconnection matrices. To assess the stability of the findings across different parcellation resolutions and strategies, we also conducted sensitivity analyses using a more fine-grained subdivision of the cortex based on the 400-parcel version of the Schaefer atlas (*26*), as well as the Glasser HCP multimodal atlas, dividing the cortex into 360 parcels based on a combination of structural and functional features (*32*).

### Atrophy mapping

To quantify regional atrophy at baseline, we generated *W*-score maps for each participant, following an established procedure (*18*, *63*). Region-wise linear regressions were first performed in the HC group to estimate the physiological effects of age, sex, intracranial volume (for subcortical volumes), and site on cortical thickness and subcortical volume values. *W*-score maps were then computed for each participant using the following formula

$$W_{i}=\frac{\text{obs}\;{\text{thickness}}_{i}\;\text{or}\;{\text{volume}}_{i}-\text{exp}\;{\text{thickness}}_{i}\;\text{or}\;{\text{volume}}_{i}}{SD_{\text{res}}}$$ (1)

where *W_i_* is the *W*-score at region *i*, obs thickness*_i_* or volume*_i_* is the observed cortical thickness or subcortical volume at region *i*, exp thickness*_i_* or volume*_i_* is the expected cortical thickness in matched HCs at region *i* given the subject’s age, sex, intracranial volume (for subcortical volumes), and site provenance [thickness/volume ∼ age + sex (+ intracranial volume) + site], and SD_res_ is the SD of the residuals in the HC group. For interpretability, *W*-scores were inverted in sign such that more positive scores indicated greater atrophy, whereas more negative scores denoted lower atrophy. Group-level cross-sectional atrophy was expressed as the effect size (Cohen’s *d*) of the difference between *W*-score maps of pwMS/CIS and HCs, assessing statistical significance with a *t* test and adjusting for multiple comparisons using false discovery rate (FDR) correction, with *P* < 0.05 indicating statistical significance.

### Spatial permutation tests

We used the ENIGMA Toolbox (v2.0.3, https://github.com/MICA-MNI/ENIGMA) in Python v3.9.0 to perform spatial correlations (*64*). As the intrinsic spatial smoothness in two given brain maps is known to potentially inflate the significance of their spatial correlation, we assessed statistical significance of spatial correlations against spatial autocorrelation-preserving null models (*65*). In particular, for cortical regions, we used spin permutation tests, where null models of overlap between cortical maps are generated by projecting the spatial coordinates of cortical data onto the surface spheres, applying randomly sampled rotations (10,000 repetitions), and reassigning regional values (*65*). The observed correlation coefficients are then compared against the null distributions determined by the ensemble of correlation coefficients comparing spatially permuted cortical maps. To compare spatial overlap between subcortical maps, we used a similar approach with the exception that subcortical labels were randomly shuffled as opposed to being projected onto spheres (*16*).

### Network-based atrophy modeling

To investigate the different mechanisms hypothesized to underlie MS-related neurodegeneration, we obtained a set of network-based maps using normative or participants’ data and assessed their spatial similarity with group-level regional atrophy. For connectivity-based mechanisms, we relied on normative structural and functional connectomes derived from 207 healthy adults sampled from the HCP (*56*, *66*) and made available by the ENIGMA toolbox (*64*). For transcriptomic analyses, we used gene expression data provided by the AHBA, a brain-wide gene expression atlas obtained from six human brain donors comprising microarray-derived measures from over 20,000 genes. Gene expression data, processed according to established recommendations (*15*) including mapping onto the Schaefer parcellations and removal of genes whose similarity across donors fell below a *r* < 0.2 threshold (leaving a total of 11,053 genes for the 100-parcel and 7584 for the 400-parcel versions), were obtained through the ENIGMA toolbox. Last, we correlated the rows of the region × gene expression matrix in pairs to generate a symmetric region × region correlated gene expression (CGE) matrix, indicating the similarity of gene expression profiles between different brain regions (*15*). To investigate the nodal stress hypothesis, we calculated regional strength centrality maps from the normative structural and functional connectomes as the sum of all cortico-cortical or subcortico-cortical edges values insisting on each node and tested their spatial correlation with GM atrophy (*16*).

For transneuronal degeneration, we tested the spatial correlation between cortical and subcortical atrophy and neighborhood atrophy maps, calculated as follows

$$A_{i}=\frac{1}{N_{i}}\sum_{j=1}^{N_{i}}a_{j}\times {\text{conn}}_{i,j},j\neq i$$ (2)

where *A_i_* is the average neighborhood atrophy of node *i*, *a_j_* is the atrophy of the *j*th neighbor of node *i*, conn*_i,j_* is the strength of connection between nodes *i* and *j*, and *N_i_* is the total number of node *i*’s first-degree neighbors. Self-connections (i.e., of a node with itself) were excluded (*j ≠ i*), while connectivity conn*_i,j_* was defined according to normative structural or functional connectomes in separate analyses (*18*). As a result, we obtained functional and structural neighborhood atrophy maps. As physical proximity and network connectivity are highly collinear (i.e., closer regions tend to be more strongly connected), we conducted additional analyses to disentangle the unique role of network connectivity in constraining atrophy patterns beyond the possible effect of anatomical proximity. Specifically, we computed a similar neighborhood atrophy map by replacing conn*_i,j_* with the reciprocal of the Euclidean distance between the regions’ centroids in MNI space. This physical proximity-weighted neighborhood atrophy map was then incorporated as a covariate in partial spatial permutation tests assessing the association between regional atrophy and connectivity-weighted neighborhood atrophy maps. To investigate whether atrophy was associated with lesion-induced disconnection, we computed a node disconnection map as the regional strength centrality (i.e., the sum of all cortico-cortical or subcortico-cortical edges insisting on each node) of the data-driven disconnectome matrix and tested its spatial correlation with GM atrophy. Last, for transcriptomic vulnerability, we tested the spatial correlation between GM atrophy and transcriptomic neighborhood atrophy maps, calculated as in (*2*) but using transcriptomic similarity defined according to the normative CGE matrix (instead of connectivity) as the weighting factor.

### Patient- and subgroup-tailored atrophy modeling

Network-based mechanisms were also assessed in individual patients and specific subgroups. In particular, for each patient, we assessed the spatial similarities between the individual *W*-score map and network-based maps, thus obtaining a correlation coefficient (*r*) and a *P* value (from spin/shuffle permutation tests with 10,000 repetitions) expressing the relevance of each hypothesized mechanism on a per-subject basis. To enable comparisons across subjects and subgroups, we transformed *r* values into Fisher’s *z* values using the inverse hyperbolic tangent function [*z* = arctanh(*r*)].

We assessed the distributions of correlation coefficients across participants and mechanisms, as well as whether they significantly deviated from zero (using one sample *t* tests), and the distributions of *P* values obtained from individual-level spatial permutation tests. Afterward, we investigated whether the different mechanisms differentially contributed to the observed atrophy patterns across distinct MRI-driven and clinically defined patients’ subgroups. Specifically, we subgrouped participants into distinct regional atrophy (i.e., *W*-score map) profiles using the HYDRA algorithm (https://github.com/sundelinustc/HYDRA) in Python v3.9.0, a nonlinear semisupervised machine learning approach for simultaneous binary classification (patients versus controls) and subtype identification (*29*). We tested different numbers of clusters (from *k* = 1 to *k* = 5) with default parameters and assessed the clustering performance by taking into account the stability of the obtained results: We used the adjusted Rand index (ARI) to quantify the similarity between clustering results across training folds in a 100-times repeated 10-fold cross-validation (CV) scheme (*29*). The clustering corresponding to the highest ARI was selected for subsequent analyses. Alternatively, we subgrouped patients based on their clinical phenotypes as ER (CIS and RRMS with <5 years of DD), LR (RRMS with ≥5 years of DD), SPMS, and PPMS (*30*). Differences in terms of *z*-values, from zero and between subgroups, were assessed in a linear model framework, correcting for multiple comparisons across mechanisms and subgroup pairs using FDR.

### Mapping disease epicenters

To identify potential disease epicenters of neurodegeneration, we performed patient-tailored connectivity-based epicenter mapping following a previously described approach (*31*). We hypothesized that an epicenter would be a region whose intrinsic connectivity pattern most strongly resembled the patient’s atrophy profile. In this light, we systematically compared seed-based (across all regions) normative structural and functional connectomes with individual atrophy (i.e., *W*-score) maps. As a result, we obtained structural and functional epicenter scores (*r* values) on a per-patient and per-region basis. After Fisher’s *r*-to-*z* transformation, we determined the significance of candidate epicenters at the group level with a one-sample, one-tailed *t* test, correcting for multiple comparisons across brain regions using FDR. The resulting statistical *t* maps (and corresponding *P* values) represented inferred structural and functional epicenter degrees across parcels at the group level.

### Sensitivity analysis using a different subtyping and staging algorithm

To assess the robustness of the identified HYDRA subtypes and disease epicenters, we applied an alternative subtyping and staging method. We used SuStaIn (https://github.com/ucl-pond/pySuStaIn) in Python v3.9.0, an unsupervised machine learning algorithm that jointly estimates disease clusters and their ordered sequence of biomarker abnormalities, providing both subtype and stage outputs (*33*). We adopted the piecewise linear *z*-score model, describing disease progression as the linear evolution of biomarkers along discrete levels of cumulative alterations, defined in terms of deviation from the healthy norm. Input features consisted of individual regional atrophy values (i.e., *W*-scores). As SuStaIn can be computationally demanding for large numbers of biomarkers, and its performance depends on a balance between sample size and number of biomarker events, we adopted a parcellation based on Glasser’s 44 cortices (*32*) and Aseg’s 14 subcortical structures (*67*), and to further reduce dimensionality, we aggregated equivalent regions from the left and right hemispheres, for a total of 29 regions. SuStaIn models up to four subtypes were trained and evaluated using 10-fold CV. The number of subtypes yielding the best balance between out-of-sample goodness of fit (log-likelihood) across CV folds, model simplicity, and biological plausibility was considered to be optimal.

### Network-based explanation of atrophy progression

Longitudinal atrophy progression was modeled in patients with at least two MRI visits. At the group level, linear mixed effects models were used to examine the change in cortical thickness or subcortical volume across follow-up visits: thickness or volume ∼1 + time + (1 + time | subject). Time since baseline visit (years) was used as fixed effect of interest, while subject-wise slope and intercept were modeled as random effects. Separate models were fitted for each parcel, with the resulting *t* map corresponding to the region-wise effect of time on cortical thickness/subcortical volume. Subject-level regional atrophy rates were estimated with individual linear models and expressed as the slope of regional thickness/volume change over time and then converted to annualized percentage change. In the same way as for cross-sectional atrophy, longitudinal estimates were multiplied by −1 such that more positive scores indicated greater atrophy progression whereas more negative scores denoted lower atrophy progression.

Using baseline information, we specified a model to explain subsequent atrophy progression in individual patients. We used GAMs, as implemented in the mgcv package’s bam function, combining data from all patients and all regions to fit a single unified model. GAMs are well suited for modeling spatiotemporal change because they can fit explanatory variables in a flexible, nonlinear fashion with safeguards against overfitting (*68*). These models can also include linear terms, random effect terms, and adjustment for spatial correlation among neighboring regions. Our model included nonlinear terms for network-based measures (i.e., normative structural strength centrality, normative functional strength centrality, baseline lesional disconnection, and functional, structural, and transcriptomic neighborhood atrophy), as well as baseline atrophy, age, follow-up duration, spatial autocorrelation (based on each region’s center of gravity coordinates), random intercepts for patients and region, and a random slope for each region’s relationship between baseline atrophy and subsequent atrophy. The linear fixed effects were sex, site, and a binary variable identifying each region as cortical or subcortical. We used the fast REML estimation method, enabling selection penalties to be added to the smooth effects. To assess the added value of network-derived metrics in explaining subsequent atrophy, we also fitted a baseline model including only nonnetwork-based explanatory variables. Comparison was made in terms of the models’ AIC and with a likelihood ratio test.
