# Supplementary material for: The role of network connectivity and transcriptomic vulnerability in shaping gray matter atrophy in multiple sclerosis
Source: Sci Adv. 2026 Nov 11;12(46):eaeg3412. doi: 10.1126/sciadv.aeg3412 (PMC13754032; doi:10.1126/sciadv.aeg3412)
Supplement: Supplementary file 1 [file sciadv.aeg3412_sm.pdf]

Science Advances
AAAS

Supplementary Materials for

The role of network connectivity and transcriptomic vulnerability in shaping gray matter atrophy in multiple sclerosis

Mar Barrantes-Cepas *et al.*

Corresponding author: Mar Barrantes-Cepas, m.barrantescepas@amsterdamumc.nl

Sci. Adv. **12**, eaeg3412 (2026)
DOI: 10.1126/sciadv.aeg3412

This PDF file includes:

Figs. S1 to S12
Tables S1 to S4

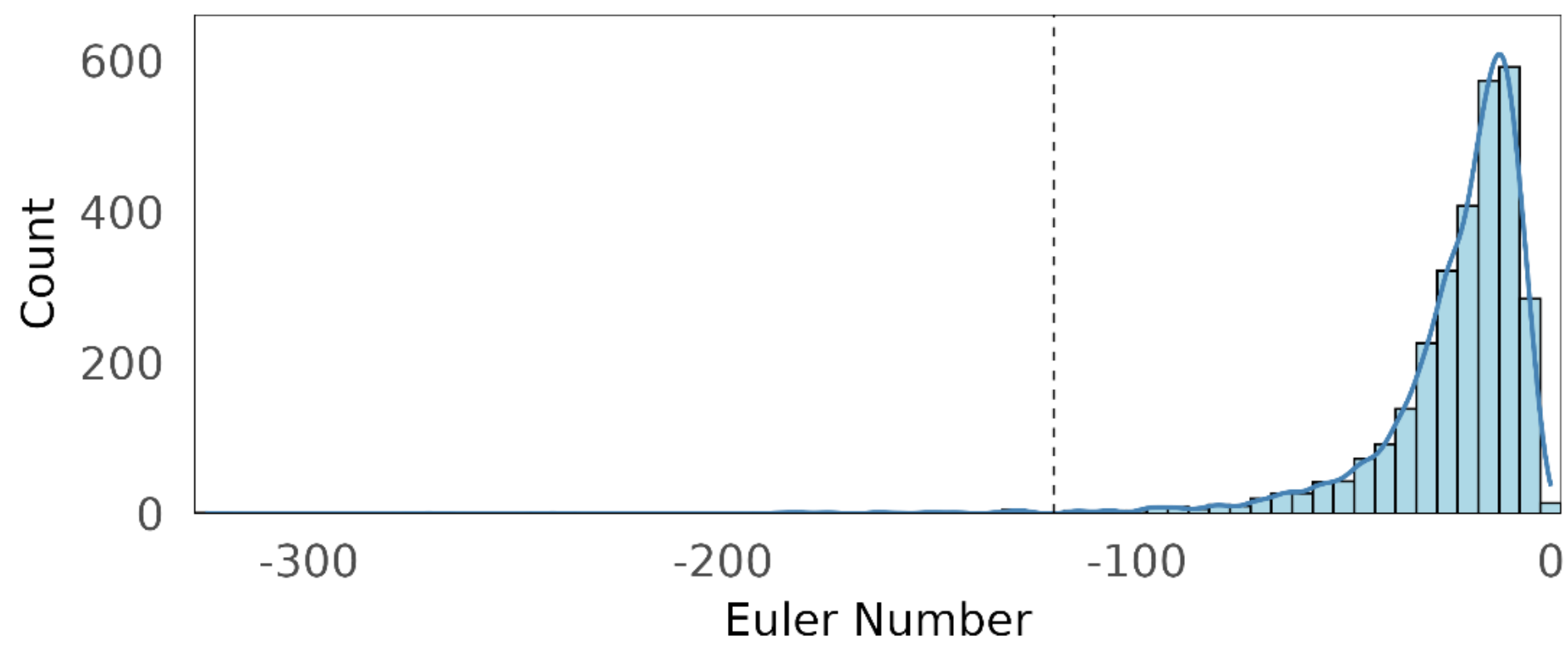

Fig. S1. FreeSurfer Quality Check. Histogram showing the distribution of Euler numbers for all processed scans, with the dashed line placed at the cutoff threshold of -120.

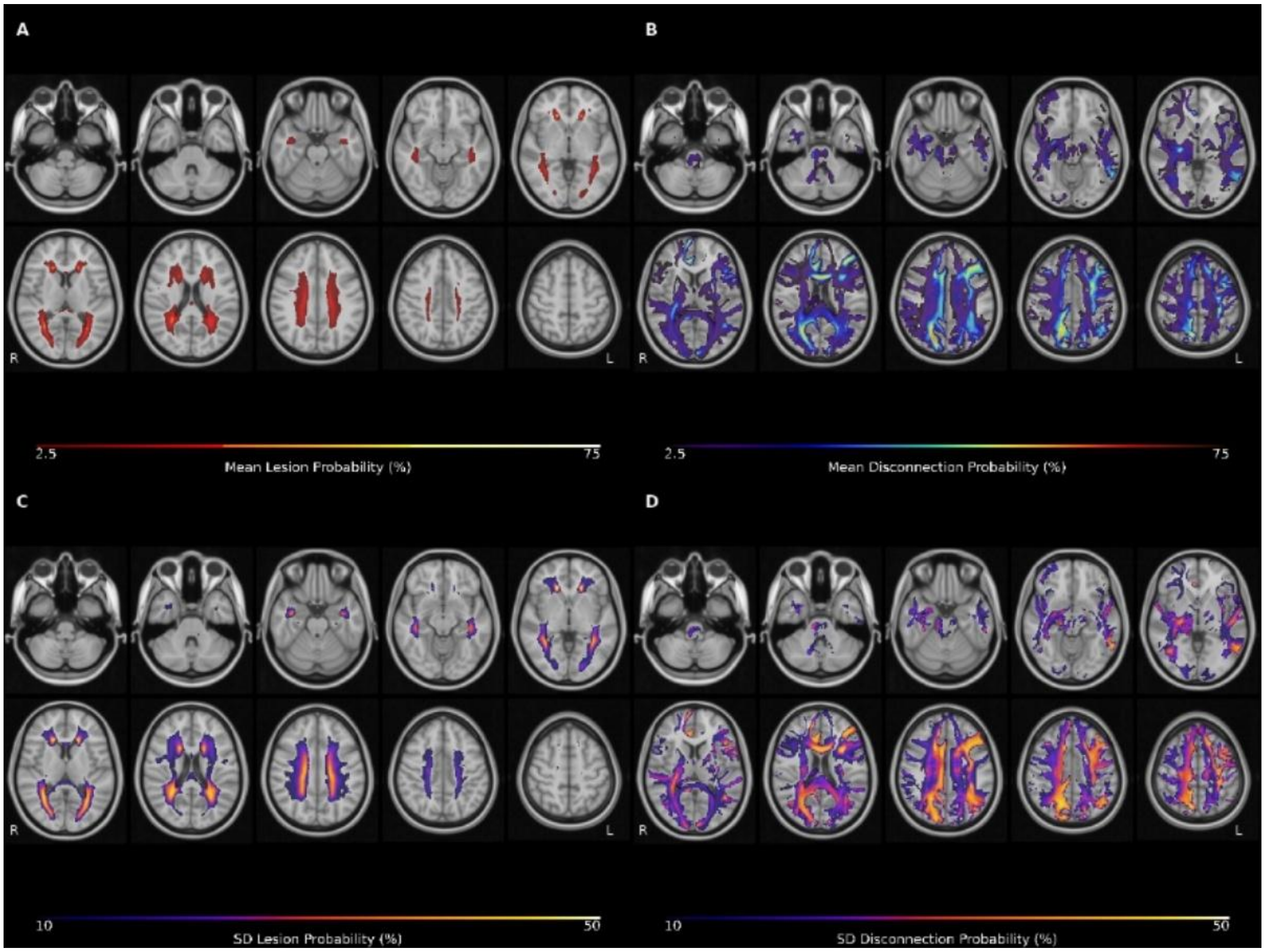

Fig S2. Lesion and disconnection probability maps. Group-level mean (A) lesion and (B) disconnection probability maps in pwMS, expressing the probabilities of each voxel containing a lesion or at least one streamline intersecting a lesion, respectively. Corresponding standard deviation (SD) maps for (C) lesion and (D) disconnection are also shown.

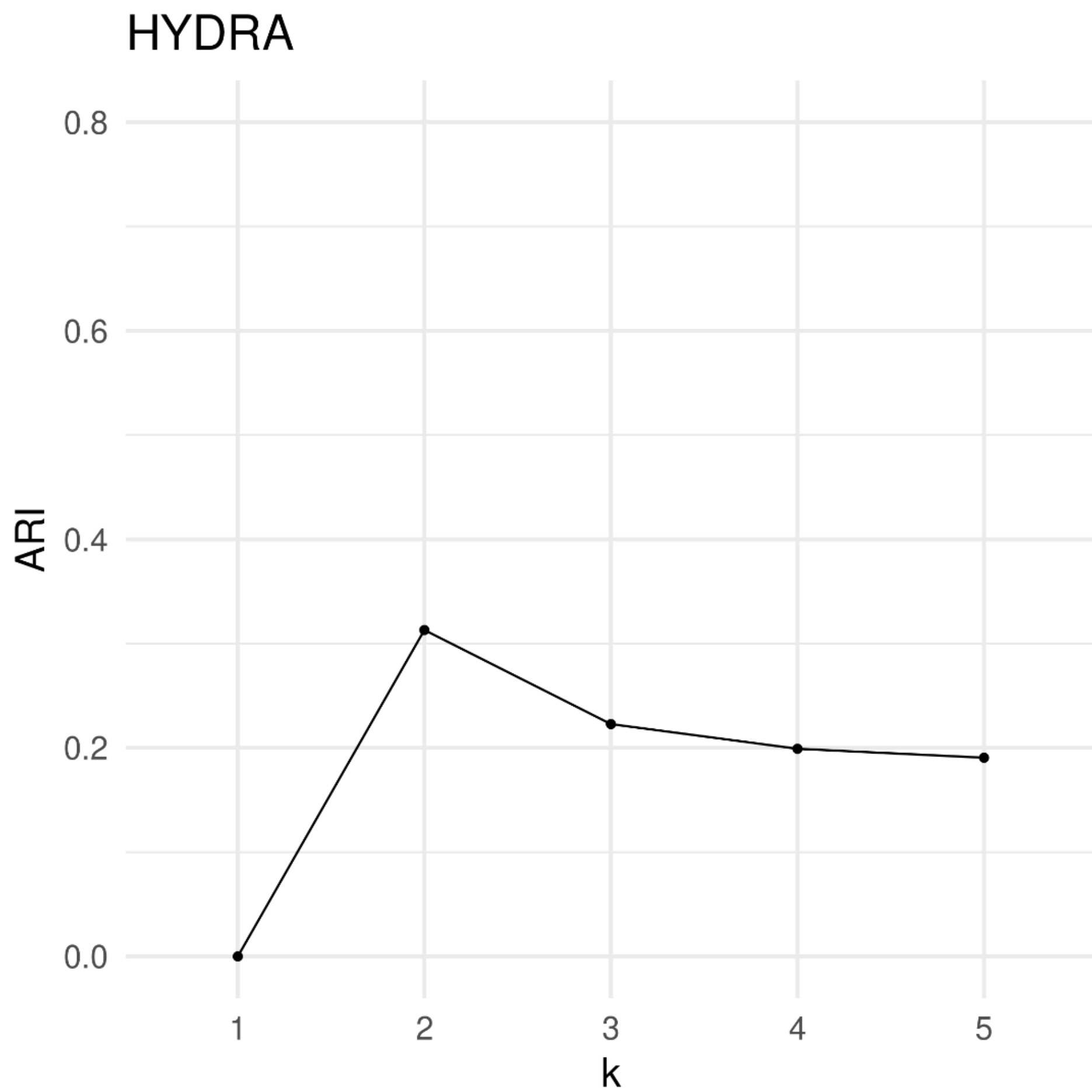

Fig. S3. Results of the data-driven clustering using HYDRA. Plot showing the stability of the identified clusters across cross-validation folds, expressed in terms of Adjusted Rand Index (ARI). We tested different numbers of clusters (from $k = 1$ to $k = 5$), with the cluster stability maximized at $k = 2$.

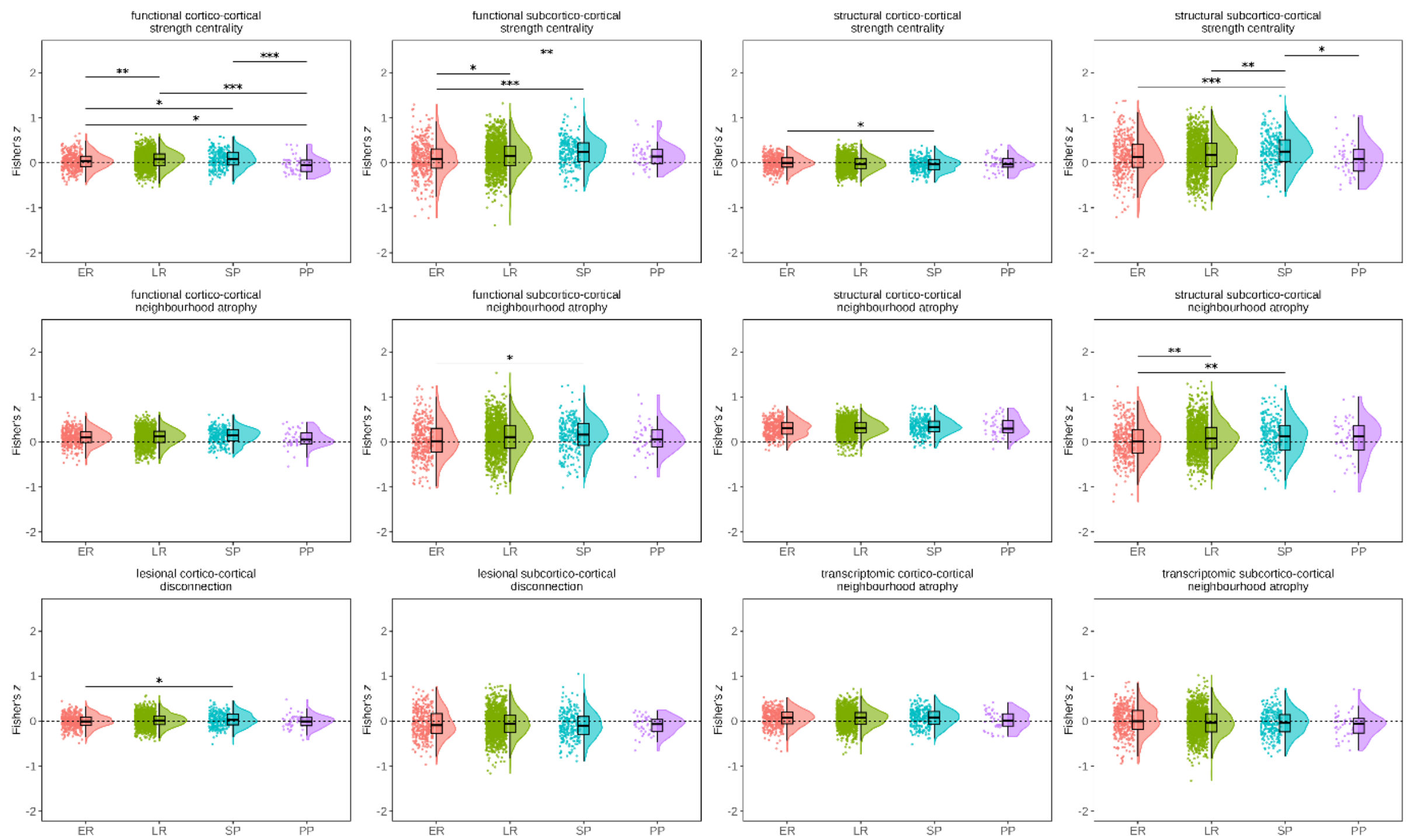

Fig. S4. Putative neurodegeneration mechanisms across clinical phenotypes. Differences between clinical phenotypes were first identified using ANOVA, followed by Tukey's post-hoc pairwise comparisons. $p < 0.05$ (*), $p < 0.01$ (**), $p < 0.001$ (***). Abbreviations: ER = early relapsing; LR = late relapsing; PP = primary progressive; SP = secondary progressive.

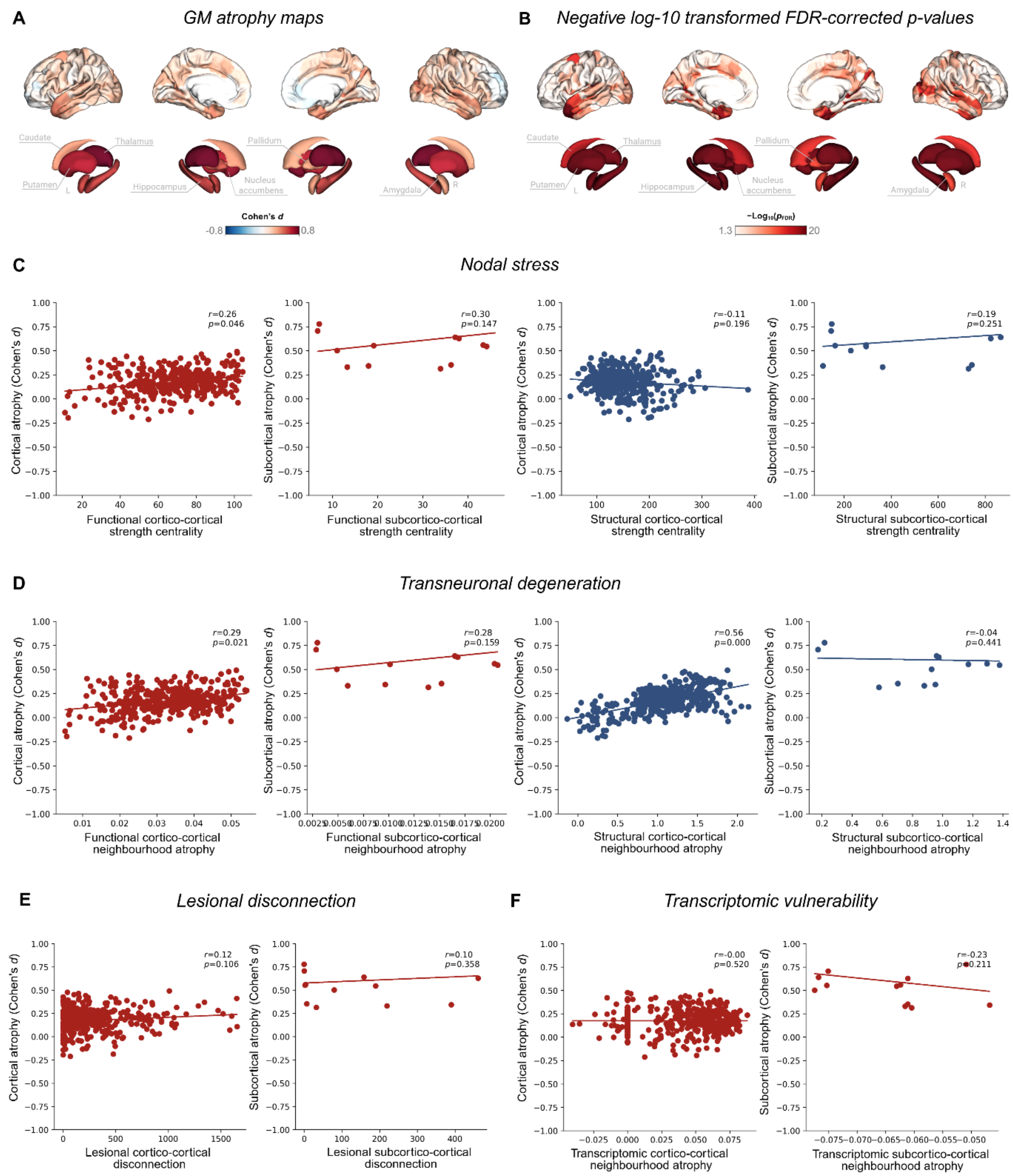

Fig. S5. Network-based atrophy mechanisms using Schaefer 400 parcellation. (A) Atrophy maps show cross-sectional differences (Cohen's *d*) in cortical thickness and subcortical volumes between pwMS/CIS and HCs, adjusting for physiological effects of age, sex, intracranial volume (for subcortical volumes), and site. (B) Negative log10-transformed FDR-corrected p-values are shown. (C) Correlation between functional and structural strength centrality and grey matter atrophy. (D) Correlation between functional and structural neighbourhood atrophy and grey matter atrophy. (E) Correlation lesion-related disconnection and (F) transcriptomic neighbourhood atrophy and grey matter atrophy.

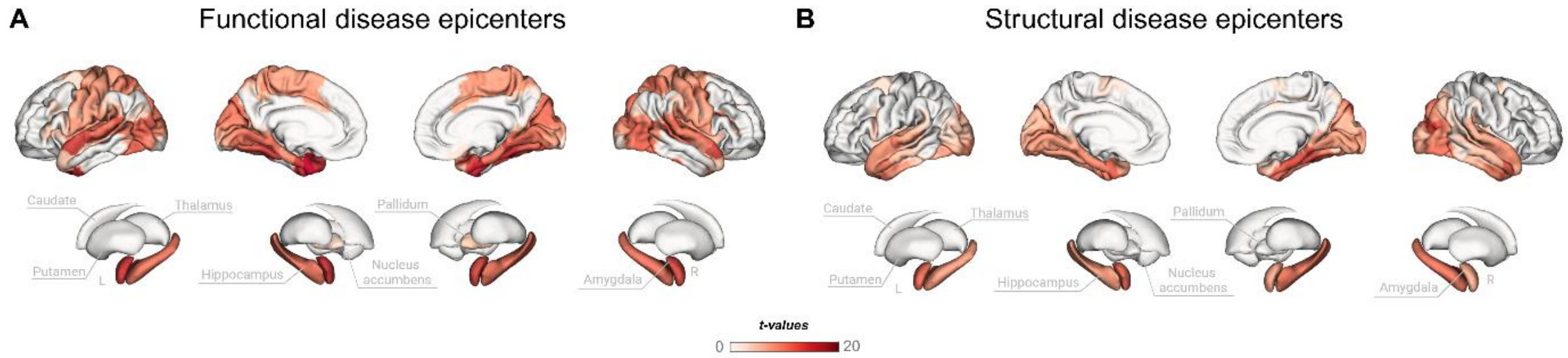

Fig. S6. Disease epicentres using Schaefer 400 parcellation. Individual-level disease epicentres were computed, Fisher *r*-to-*z* transformed, and assessed for group-level significance using one-tailed *t*-tests with FDR correction. Shown are regions exhibiting significant effects, with maps displaying the corresponding *t*-values.

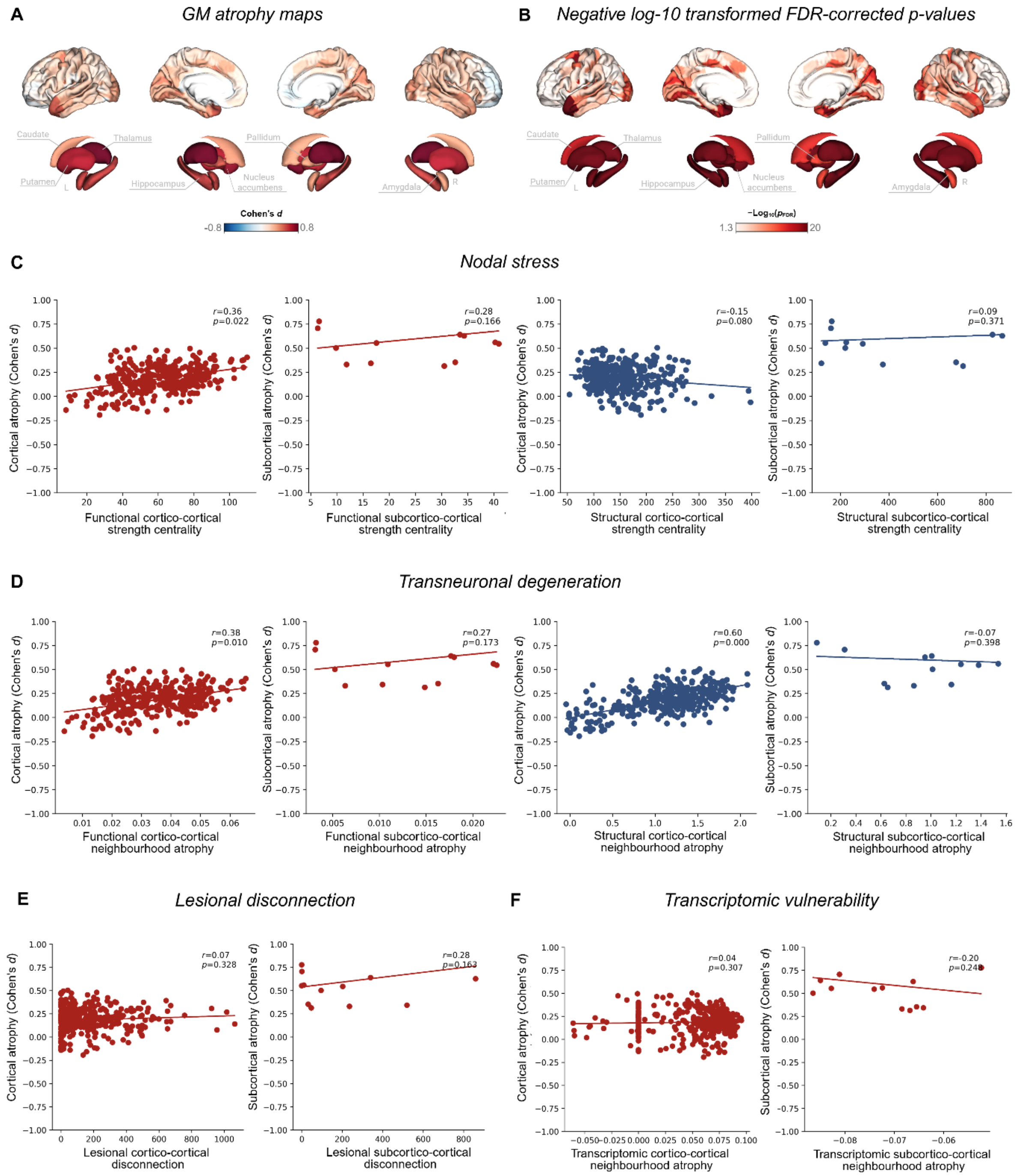

Fig. S7. Network-based atrophy mechanisms using Glasser parcellation. (A) Atrophy maps show cross-sectional differences (Cohen's *d*) in cortical thickness and subcortical volumes between pwMS/CIS and HCs, adjusting for physiological effects of age, sex, intracranial volume (for subcortical volumes), and site. (B) Negative log10-transformed FDR-corrected p-values are shown. (C) Correlation between functional and structural strength centrality and grey matter atrophy. (D) Correlation between functional and structural neighbourhood atrophy and grey matter atrophy. (E) Correlation lesion-related disconnection and (F) transcriptomic neighbourhood atrophy and grey matter atrophy.

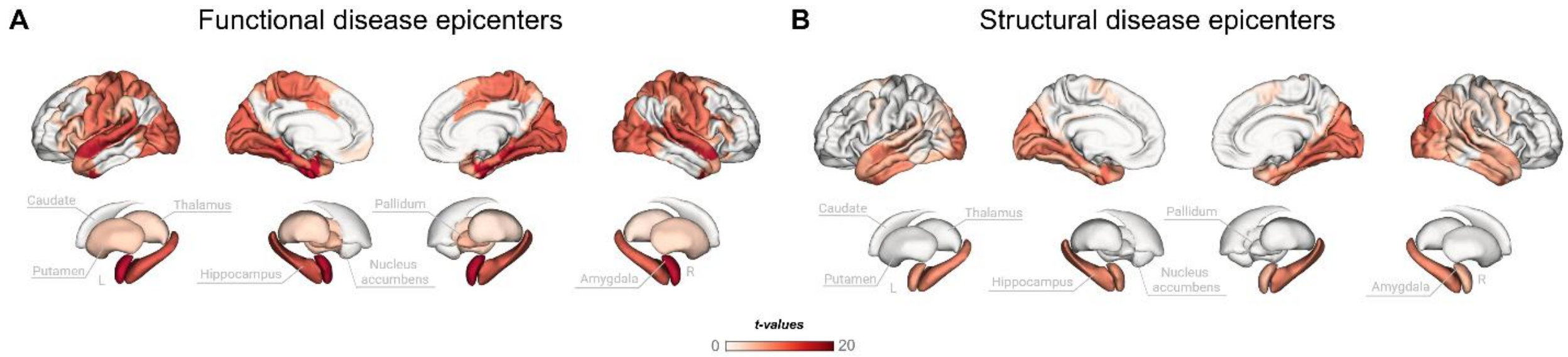

Fig. S8. Disease epicentres using Glasser parcellation. Individual-level disease epicentres were computed, Fisher *r*-to-*z* transformed, and assessed for group-level significance using one-tailed *t*-tests with FDR correction. Shown are regions exhibiting significant effects, with maps displaying the corresponding *t*-values.

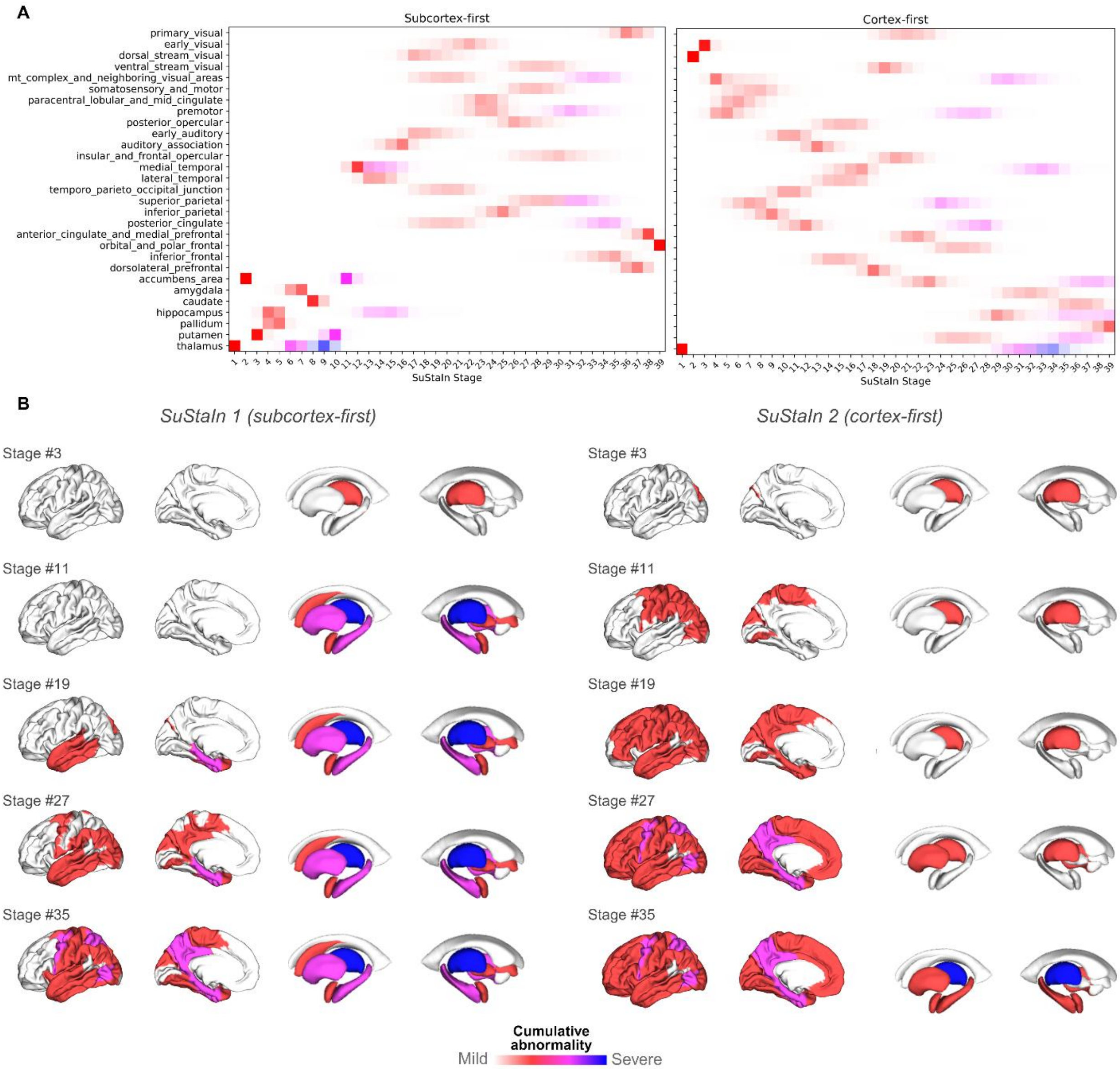

Fig. S9. SuStaIn subtypes and disease staging. (A) Positional variance diagrams (PVDs) for the two SuStaIn subtypes (SuStaIn1, SuStaIn2), showing the most probable order of regional grey matter atrophy events across disease stages, based on the Glasser 22-region parcellation. (B) Surface maps of regions affected at stages 3, 11, 19, 27, and 35 for each subtype, with color intensity proportional to cumulative atrophy probability at each stage.

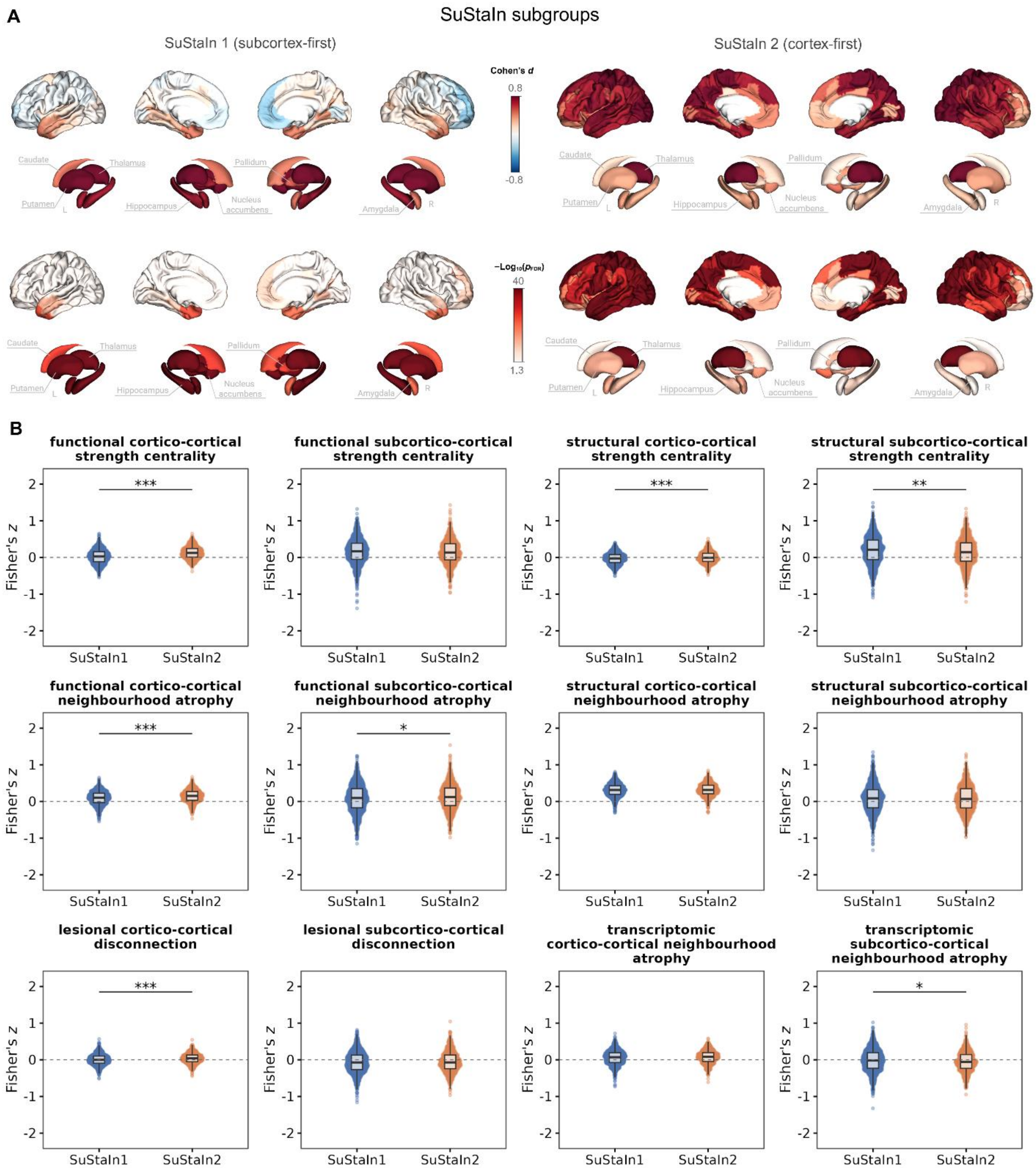

Fig. S10. Putative neurodegeneration mechanisms across SuStaIn subgroups. (A) Atrophy maps showing effect sizes (Cohen's *d*) and negative log10-transformed FDR-corrected *p*-values for SuStaIn groups. (B) Differences between clinical phenotypes were first identified using ANOVA, followed by Tukey's post-hoc pairwise comparisons. $p < 0.05$ (*), $p < 0.01$ (**), $p < 0.001$ (***).

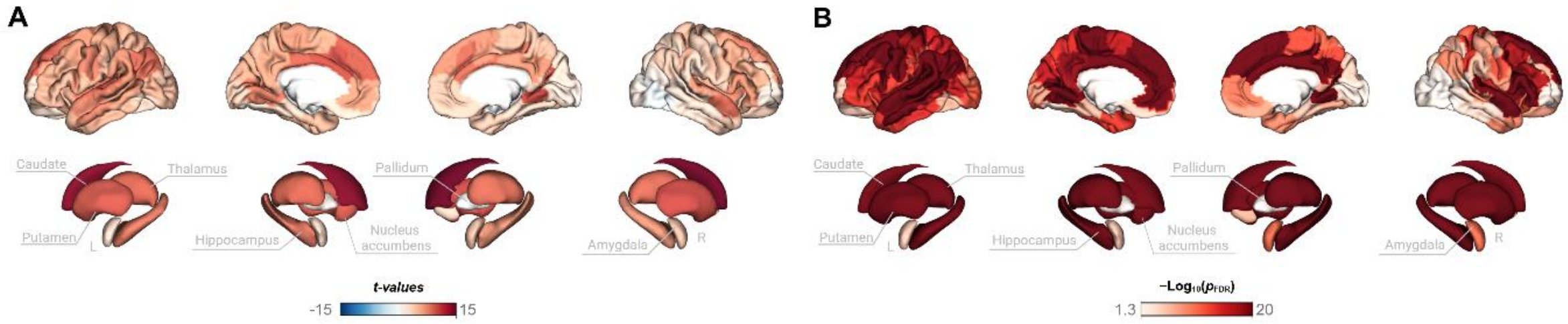

Fig. S11. Longitudinal atrophy profiles. (A) Atrophy maps show longitudinal atrophy (*t*-values) in cortical thickness and subcortical volumes. Longitudinal atrophy progression was modelled in patients with at least two MRI visits. (B) Negative log10-transformed FDR-corrected *p*-values are shown.

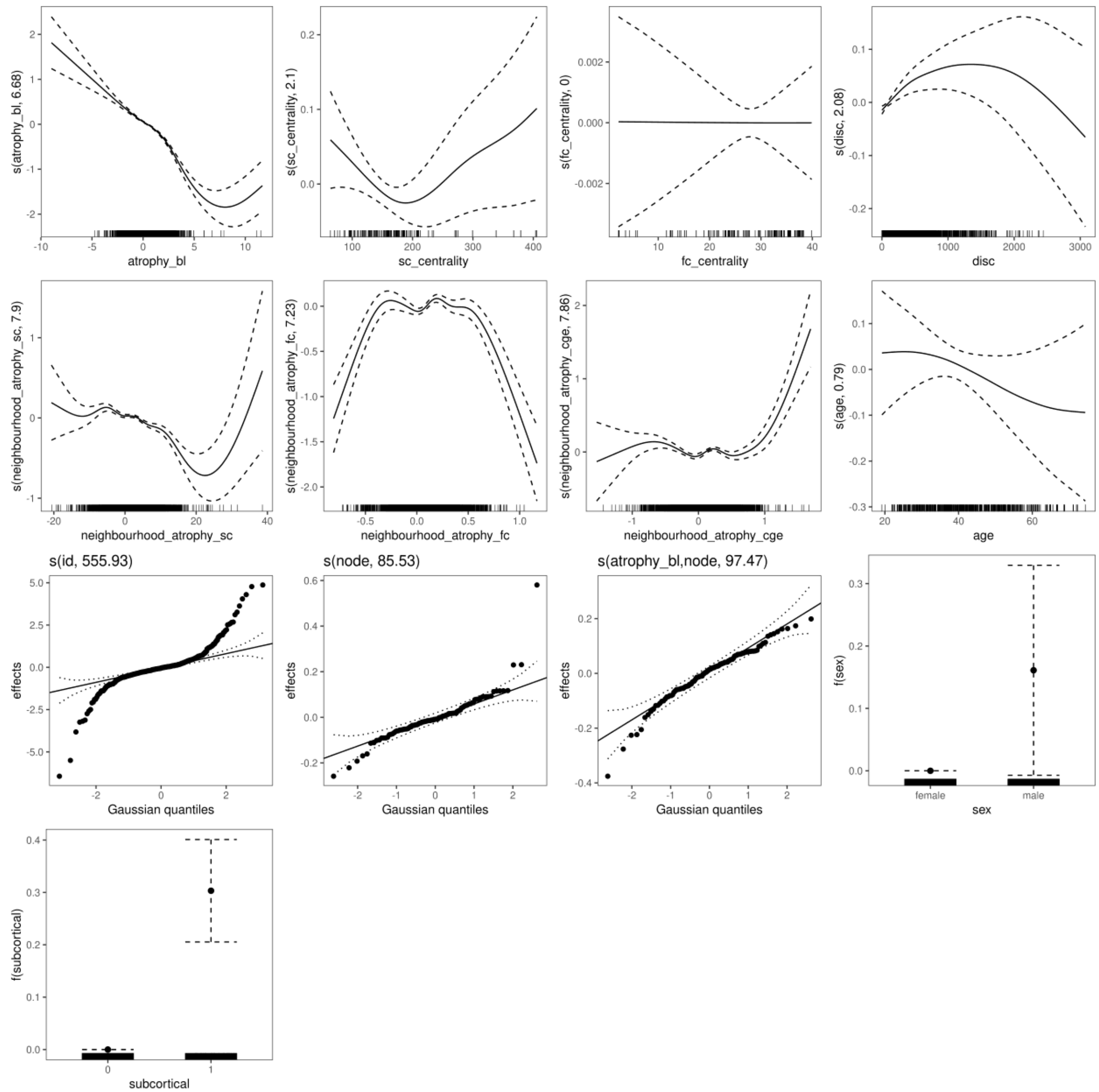

Fig. S12. Network-based model for explaining longitudinal atrophy. Effect plots for the explanatory variables of interest, where dashed lines correspond to 95% confidence interval of the fit.

Centre	Amsterdam		Graz		Prague	
**Field strength**	3 Tesla		3 Tesla		3 Tesla	
**Vendor**	GE		Siemens		Siemens	
**Model**	Discovery MR750		Prisma		Skyra	
**Years**	2014-2023		2021-2022		2012-2022	
**Modality**	**T1w**	**FLAIR**	**T1w**	**FLAIR**	**T1w**	**FLAIR**
**Voxel dimensions (mm^3)**	0.94x0.94x1	0.98x0.98x1.2	1x1x1	1x1x1	1x1x1	1x1x1
**TR (ms)**	7.8	8000	1900	5000	2300	5000
**TE (ms)**	3	125	2.7	393	3	397
**TI (ms)**	450	2350	900	1800	900	1800
**FA (°)**	12	-	9	120	9	120
**Slices, Orientation**	172, sag	128, sag	176, sag	176, sag	176, sag	176, sag

Table S1. MRI acquisition protocols. Abbreviation: FLAIR = fluid-attenuated inversion recovery; mm = millimetre; ms = milliseconds; T1w = T1-weigthed; TE = echo time; TI = inversion time; TR = repetition time; FA = flip angle; sag = sagittal.

**Reason for exclusion**	***N***
FreeSurfer failed	9
FreeSurfer Quality Control (based on Euler's number)	31

Table S2. Number and reason for excluded scans. Exclusion reasons are summarized for all participants who were not included in the final analysis. Numbers represent the count of sessions excluded for each reason.

	HYDRA1 widespread atrophy	HYDRA2 spared cortex	*p*-value
N	997	966	
Age [years], mean±SD	44.4±9.8	46.4±9.8	<0.001[a]
Female, *N* (%)	628 (63.0%)	747 (77.3%)	<0.001[b]
**Disease characteristics**			
Phenotype, *N* (%)			0.016[b]
CIS	29 (2.9%)	28 (2.9%)	
RRMS	802 (80.4%)	818 (84.7%)	
SPMS	146 (14.6%)	96 (9.9%)	
PPMS	20 (2.0%)	24 (2.5%)	
Disease duration [years], mean±SD	14.4±9.2	14.6±9.2	0.69[a]
EDSS, median (range)	2.5 [0 – 8.0]	2.5 [0 – 8.5]	0.19[a]
DMT, *N* (%): yes/no	778 (78%) / 219 (22%)	727 (75.3%) / 239 (24.7%)	0.16[b]
TLV [mL], median (IQR)	4.35 [1.37 – 11.67]	2.68 [0.91 – 7.32]	<0.001[a]
BPF, mean±SD	0.74±0.05	0.75±0.05	<0.001[a]

Table S3. Demographic, clinical and MRI characteristics of HYDRA subgroups. Baseline characteristics of included data-driven HYDRA groups. Abbreviations: BPF = brain parenchymal fraction, CIS = clinically isolated syndrome, DMT = disease-modifying treatment, EDSS = Expanded Disability Status Scale, IQR = interquartile range, mL = millilitres, PPMS = primary-progressive multiple sclerosis, RRMS = relapsing-remitting multiple sclerosis, SD = standard deviation, SPMS = secondary-progressive multiple sclerosis, TLV = total lesion volume. [a]Permutation *t*-test (n = 10,000); [b]Chi-square test.

	SuStaIn1 subcortex-first	SuStaIn2 cortex-first	*p*-value
N	1277	686	
Age [years], mean±SD	46.2±9.9	43.6±9.5	<0.001[a]
Female, *N* (%)	944 (73.9%)	431 (62.8%)	<0.001[b]
**Disease characteristics**			
Phenotype, *N* (%)			0.015[b]
CIS	33 (2.6%)	24 (3.5%)	
RRMS	1030 (80.7%)	590 (86.0%)	
SPMS	183 (14.3%)	59 (8.6%)	
PPMS	31 (2.4%)	13 (1.9%)	
Disease duration [years], mean±SD	15.4±9.5	12.9±8.4	<0.001[a]
EDSS, median (range)	2.5 [0 – 8.5]	2.0 [0 – 8.0]	<0.001[a]
DMT, *N* (%): yes/no	991 (78%) / 286 (22%)	514 (74.9%) / 172 (25.1%)	0.20[b]
TLV [mL], median (IQR)	3.96 [1.27 – 10.86]	2.66 [0.78 – 6.76]	<0.001[a]
BPF, mean±SD	0.74±0.04	0.76±0.06	<0.001[a]

Table S4. Demographic, clinical and MRI characteristics of SuStaIn subgroups. Baseline characteristics of included data-driven SuStaIn groups. Abbreviations: BPF = brain parenchymal fraction, CIS = clinically isolated syndrome, DMT = disease-modifying treatment, EDSS = Expanded Disability Status Scale, IQR = interquartile range, mL = millilitres, PPMS = primary-progressive multiple sclerosis, RRMS = relapsing-remitting multiple sclerosis, SD = standard deviation, SPMS = secondary-progressive multiple sclerosis, TLV = total lesion volume. [a]Permutation *t*-test (n = 10,000); [b]Chi-square test.
